# Climatic niche characteristics of native and invasive *Lilium lancifolium*

**DOI:** 10.1038/s41598-019-50762-4

**Published:** 2019-10-04

**Authors:** Sonia Herrando-Moraira, Neus Nualart, Albert Herrando-Moraira, Mi Yoon Chung, Myong Gi Chung, Jordi López-Pujol

**Affiliations:** 1 Botanic Institute of Barcelona (IBB, CSIC-ICUB), Barcelona, 08038 Catalonia Spain; 2 Polytechnic University of Catalonia, Terrassa, 08222 Catalonia Spain; 3 Research Institute of Natural Science (RINS), Gyeongsang National University, Jinju, 52828 Republic of Korea; 4 Division of Life Science and RINS, Gyeongsang National University, Jinju, 52828 Republic of Korea

**Keywords:** Plant sciences, Ecology

## Abstract

One of the topics currently under discussion in biological invasions is whether the species’ climatic niche has been conserved or, alternatively, has diverged during invasions. Here, we explore niche dynamic processes using the complex invasion history model of *Lilium lancifolium*, which is the first tested case of a native species (Korea) with two hypothesized spatial (regional and intercontinental) and temporal arrivals: (1) as an archaeophyte in East Asia (before AD 1500); and (2) as a neophyte in Europe, North America, Australia, and New Zealand (after AD 1500). Following a niche examination through both environmental and geographical spaces, the species in the archaeophyte range has apparently filled the ancestral native niche and, rather, would have increased it considerably. The species as a neophyte shows a closer climatic match with the archaeophyte range than with the native one. This pattern of niche similarity suggests that the neophyte range was probably colonized by a subset of archaeophyte propagules adapted to local climate that promoted the species’ establishment. Overall, niche conservatism is proposed at each colonization step, from native to archaeophyte, and from archaeophyte to neophyte ranges. We detected signals of an advanced invasion stage within the archaeophyte range and traces of an early introduction stage in neophyte ranges.

## Introduction

Biological invasions are one of the most complex ecological processes, in which many factors such as introduction history, species invasiveness, or community invasibility, determine the success of the species’ establishment^1^. Moreover, this process is currently being accelerated in short time frames due to globalization, the loss of natural biogeographic barriers, or accidental/deliberate releases^2,3^. These worldwide species invasions represent natural experiments to explore rapid ecological changes that could take place during the colonization process of novel environments^4^.

In ecological niche research, one of the main topics of debate is whether the species’ niche has been conserved or has diverged during the invasion process^5,6^. The conservation of the ancestral niche in the invaded ranges implies that climates between the native and invaded areas are analogous, and thus the likelihood of a successful invasion is high due to the pre-adapted condition of the introduced pool (climate match hypothesis^7^). Accordingly, the hypothesis of niche conservatism has been extensively used as a proxy to predict the invasion risk of introduced species^8,9^. However, niche conservatism is not always a universal rule applicable to all biological invasions, since the species can grow, survive, or adapt to novel environmental conditions that do not exist in the source area, or that they are available but not yet occupied. In such cases, it can be stated that a “niche shift” has occurred (see review in^10^). In a recent review of more than 800 terrestrial plant species, Atwater *et al*.^11^ demonstrated that niche shifts are more common than previously reported in introduced plants^12^. Moreover, Atwater *et al*.^11^ found that, among the intercontinental invasions, cultivated and woody species are more prone to exhibit climatic niche shifts than species with other biological traits. Several mechanisms, which may act in concert, have been traditionally attributed to niche shifts: (1) modifications of biotic interactions, such as low predation or competition pressure^13,14^; (2) dispersal limitations due to geographical barriers^15^; and (3) a partial filling of the global native climates^16^. Nevertheless, it should be noted that these factors may actually correspond to a “non-evolutionary niche shift”, which means that the niche change is likely the result of environmental heterogeneity (i.e. non-analogue climates) between native and invaded ranges^17^. A real evolutionary change in the fundamental niche would be proposed for those cases in which similar climatic background exists in native–introduced areas and the species is actively selecting different climatic spaces^18^. However, a true “evolutionary niche shift” is more difficult to test and corroborate empirically^19^.

For a proper assessment of possible niche changes, it is crucial to identify the stage of the invasion, i.e. the equilibrium degree between invader and environment^20^. The observed niche dynamics can be really different depending on the current invasion stage: dispersal from the source area (transport), establishment of the initial propagules (introduction), growth and reproduction (establishment), or expansion-dispersal into new areas (spread)^21,22^. As a general pattern, in early stages of an invasion, when the species is still in a non-equilibrium with the environment, one can expect to find traces of bottlenecks and founder effects^23,24^, long lag-phases or colonization time lag^25^, pre-adaptations from the original sources^26^, or even variations due to new selective pressures^27^. Conversely, in advanced colonization phases, the progressive admixture of introduced lineages of a given species may lead to the restoration of its genetic variability^28^, an increase in its distribution area, and in some cases, its becoming an invasive species causing adverse ecological impacts^29^.

To test for climatic niche shifts, several methodological tools have been developed in the last decades both in the environmental (E-space) and geographical space (G-space) (see figure 3 in^10^). All these methods, however, should be used with caution, as a given species could have not access to all possible environmental conditions (e.g. restricted by barriers, dispersal disequilibrium, or negative interactions); i.e. the fundamental niche could not be equated to the realized niche^30^.

In the case of the G-space analyses, one of the most widely and popularly used approaches is the reciprocal species distribution modeling (SDM), where the models are calibrated in the native range and then projected into the introduced one, and vice versa^23,31–33^. Thereby, when the niche is conserved during the invasion process, spatial predictions calibrated in the native range show suitable signals in the current introduced ranges. In this case, reciprocal SDMs aid in predicting possible areas at risk or that are susceptible to invasion^31^. Alternatively, when the niche has diverged, the native model fails to predict the areas of introduction. The reciprocal SDMs are currently, however, quite controversial due to several methodological limitations: (1) they are based on the principle of niche conservatism^34^; (2) they do not account for dispersal constraints and biotic interactions^35^; (3) they do not consider the available climatic space in both ranges^12^, which can lead to detecting niche differences due to different climatic space available^10^; (4) they do not quantify the degree of realized niche overlap or niche dynamics^36^; and (5) they do not determine the climatic direction in which the niche shifts occur^11^.

Regarding the E-space evaluations, some of the abovementioned G-space shortcomings have been overcome. The ordination method, based on principal component analysis (PCA), is being increasingly used for niche comparisons in the E-space, mainly under the framework developed by Broennimann *et al*.^36^. Certainly, a remarkable advantage of this method is the implementation of a kernel density function to the presence records in order to smooth the occurrence density by their prevalence in the climatic space generated from the PCA. In this way, the potential sampling bias produced by the impossibility of collecting all occurrences from the entire distribution of species is highly minimized^36^. Thus, if certain areas are undersampled due, for instance, to data gaps of some world regions^37^, we will not get an overrepresented niche that can lead to miscalculations of niche dynamics and overlap values. Furthermore, recent refinements of this method account for outlining multiple and simultaneously realized niches across the global climatic space from the PCA^38^, which is undoubtedly a great improvement for cases of more than one introduction to new distribution ranges.

*Lilium lancifolium* Thunb. (=*L. tigrinum* Ker Gawl.) constitutes a polyploid complex involving diploid (2*n* = 2*x* = 24) and triploid (2*n* = 3*x* = 36) forms^39–43^, which are morphologically hardly distinguishable^44^ (see Chung *et al*.^42^ for the full description of the plant). The triploid cytotype is completely sterile because flowers do not produce capsules^40^; however, triploids have vigorous vegetative reproduction through bulbils, which are formed along the whole stem. Although the origin of the triploid cytotype of *L. lancifolium* has been a matter of debate during the past half century, most of the studies carried out during the last decade strongly support an autopolyploid origin, including classical and modern cytogenetic approaches^45–47^ and genetic diversity studies^42,48^. The diploid cytotype, despite being locally common, has a very narrow distribution, occurring in western and southern coastal areas of the Korean Peninsula (including Jeju Island and surrounding smaller islands), and in the Japanese islands of Tsushima and Iki^41,43^, although it has also been once cited in Russia, near Vladivostok, in Primorsky Region^49^. Compared to the relatively limited distribution of diploid *L. lancifolium*, triploid forms would have a much broader distribution occurring in the inland areas of the Korean Peninsula, in coastal areas of eastern Korea, and in Jeju and Tsushima islands^41,43^.

In other parts of East Asia, populations of *L. lancifolium* have been observed in large portions of China, in the main Japanese islands (Hokkaido, Honshu, Kyushu, and Shikoku), and in the south-eastern tip of the Russian Far East (Primorsky Krai and Sakhalin Oblast). These populations have been generally assumed as belonging to the triploid cytotype^40,42^, although cytogenetic data are far from being complete (Appendix B, Table S1 in Electronic Supplementary Material). In China, Japan (except Tsushima), and the Russian Far East, *L. lancifolium* is likely an “archaeophyte” (defined as those non-native plants introduced before AD 1500^21^), given the fact that it has been widely cultivated in China and in Japan (including Hokkaido, the northernmost island of Japan) during the last one or perhaps two millennia^50–53^ (but see^54^) for its edible bulbs and medicinal uses^40,55–57^. The significance of *L. lancifolium* in the ancient Chinese culture is exemplified by its representation in the cave no. 130 of Mogao (Dunhuang, Gansu Province), which dates from the High Tang period (705–781 AD^58^). In contrast, *L. lancifolium* has been rarely cultivated in Korea and never in Tsushima Island^40^ (JM Chung, Korea National Arboretum, pers. comm.), where it should be regarded as a native plant.

Outside East Asia, *L. lancifolium* has been observed growing wild (either as casual or naturalized) within a few regions, including eastern North America, northern Europe, and Oceania (Australia and New Zealand), often as garden escapes. In these non-Asian areas, the plant could be regarded as a “neophyte” (species introduced after AD 1500 according to Pyšek *et al*.^21^), since the plant did not arrive in Europe until the late 17th century. According to Rushing^53^, *L. lancifolium* was firstly introduced in Europe in 1684 from Japan by Engelbert Kaempfer of the Dutch East India Company. It seems, however, that the plant did not reach western gardens until its second introduction in Europe in 1804 from Guangzhou (Canton) in China by William Kerr^59^. It was introduced somewhat later into North America (in the 1830s) where it soon became a popular garden plant^53^.

Regarding its introduction in these neophyte areas, *L. lancifolium* has often been recorded as a garden escape in North America; indeed, it was already mentioned as escaping in 1856 by the celebrated American naturalist HD Thoreau in Massachusetts^60^. In the north-eastern portion of the United States of America and in SE Canada it has become naturalized, usually occurring in ruderalized places (roadsides, railways, abandoned or vacant lots in urban areas, cemetery prairies, etc.^61^). The species, indeed, seems to be still expanding in North America, as it has recently naturalized in Texas^62^. In Oceania the species, in contrast, seems to behave already as an aggressive weed. In New Zealand, it is listed as an environmental weed at national level^63^, with the first records dating from the 1950s^64^. In Australia, *L. lancifolium* is also recognized at nation level as an invasive^65,66^; it was first recorded as naturalized in Victoria in 1985^67^, and later in 2004 in New South Wales^68^. In Europe, the species is relatively widely naturalized in Fennoscandia and the Baltic states (e.g.^69–71^). It has also been reported in Austria as an adventive^71,72^, although it seems that this species at present only occurs in this country as cultivated (C Tschisner, Inatura Museum, Austria, pers. comm.). The species has also been cited in central Italy^73^ even though these authors are not sure of its spontaneity, and in the mountains near Tirana, Albania^74^.

The present study is focused on characterizing the niche of *L. lancifolium* separately by their non-native ranges that have been reported (and that we are tentatively treating) as archaeophyte and neophyte ones. As far as we know, there are no published studies doing so. We would expect to detect signals more associated with a successionally advanced introduction in the case of archaeophyte area and, on the other hand, traces of an early introduction stage for the neophyte ranges. This worldwide species’ expansion in different time frames is used here as a model: (1) to outline possible colonization routes from niche similarity comparisons, focused on testing whether neophyte ranges could have derived from the native or from the archaeophyte areas; (2) to explore whether climatic niche has been conserved or has diverged through years and continents; and (3) to address the current niche dynamics of each distribution region.

## Methods

### Study areas

**Figure 1:**
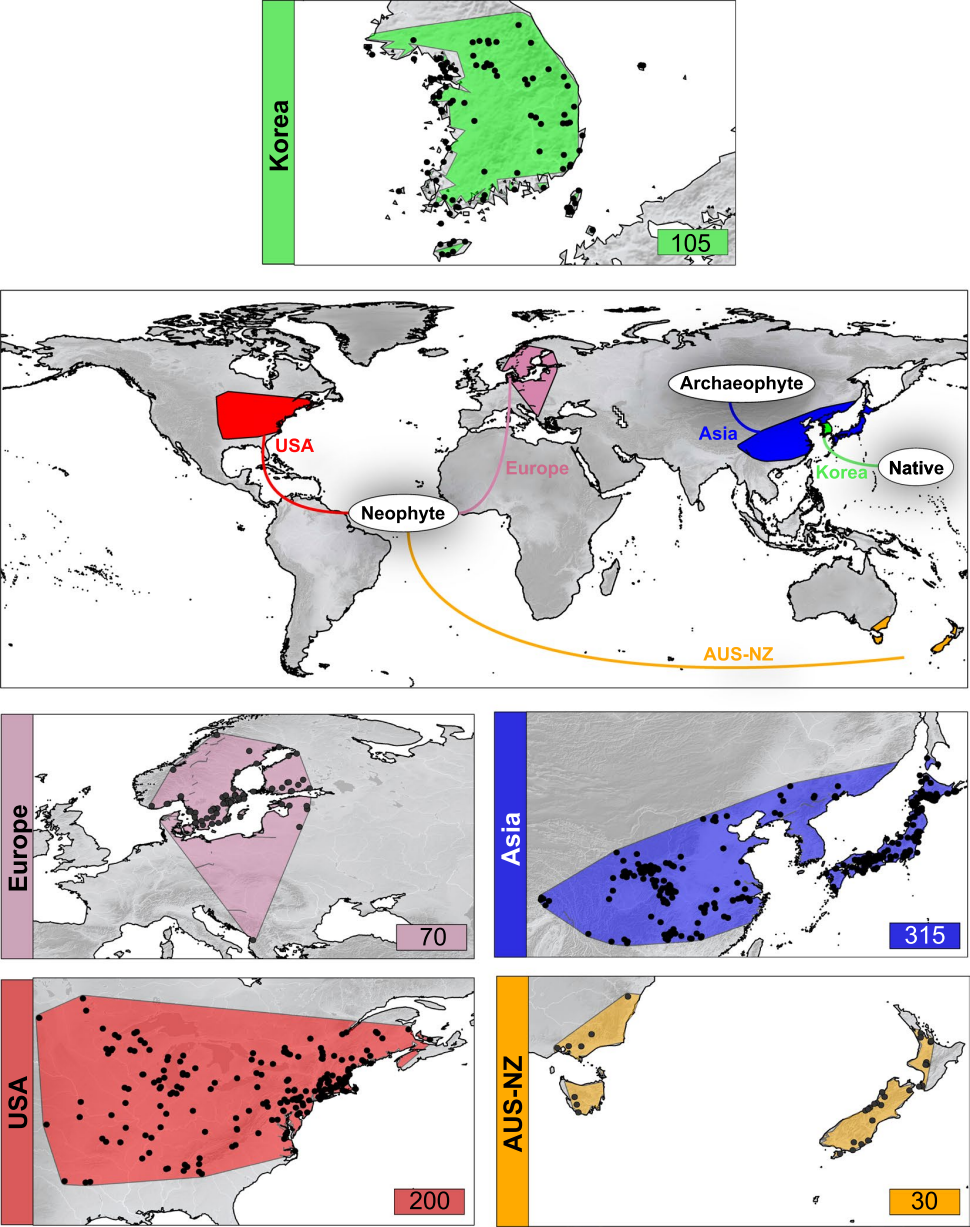
Global representation of distribution areas of *Lilium lancifolium* as native, archaeophyte, and neophyte. For each of the ranges, the occurrences are represented in black dots, and environmental background in its corresponding color is shaded. The number of total presence records used is also shown.

According to the distribution of *L. lancifolium*, five geographical backgrounds have been considered (Fig. 1). The native area is limited to the Korean Peninsula and surrounding islands (also including Tsushima in Japan), where diploid and triploid cytotypes occur. As no niche divergence has been observed between these two cytotypes^42^, all the occurrences of this area have been treated together regardless of their ploidy level and hereafter referred to as “native”. The archaeophyte area comprised all the Asian occurrences, basically in China, Japan, and the Russian Far East (including the diploid locality in Primorsky Region identified by Probatova *et al*.^49^), and are hereafter referred to as “ASIA” or “archaeophyte”. Despite the fact that some populations or part of these ranges could be of recent origin, for practical reasons we are treating all the occurrences in these countries as archaeophytes. Three “neophyte” areas have been considered: (1) the eastern half of USA and Canada (hereafter “USA-CA”); (2) the southeastern corner of Australia and New Zealand (hereafter “AUS-NZ”); and (3) Europe (hereafter cited as “EUR”), comprising the Nordic countries of Finland, Norway, and Sweden but also Lithuania, Estonia, and Albania.

### Occurrence data and climatic variables

The current distribution information was obtained from specimens deposited in herbaria—included those from China (through the Chinese Virtual Herbarium platform; https://www.cvh.ac.cn), Japan (TUS and KYO), and Korea (SNU), from presence records included in the Global Biodiversity Information Facility (GBIF, https://www.gbif.org), from personal communications (see acknowledgements), and from relevant literature (such as local floras and floristic surveys), including gray literature and websites. Only citations of wild occurrences have been taken into account, although information regarding the degree of naturalization (i.e. casual, naturalized, or invasive) is seldom available. Outlier occurrences or those locations from non-authoritative sources were thoroughly validated one by one, keeping only those with an image showing the individuals in the wild or on herbaria sheets. A complete list of all the sources used to obtain records of *L*. *lancifolium* is found in Appendix A (Electronic Supplementary Files). Although many records of *L. lancifolium* did not specify latitude and longitude, we were able to geo-reference some cases that had detailed descriptions of the localities. Those occurrences with vague descriptions (e.g. counties, provinces, or mountain ranges) that did not allow the use of an appropriate pixel resolution (2.5 arc-min, ca. 5 × 5 km) were discarded. Although finer resolutions are possible for some occurrences (e.g. 30 arc-sec), these may not be appropriate, given uncertainties associated with geo-referencing approximate localities (a common situation with old herbarium records, especially for remote areas) or with geo-reference errors. For the georeferenced localities, coordinates were checked to ensure their reliability. In order to reduce sampling bias and spatial autocorrelation, the presence dataset composed of ca. 800 occurrences was filtered following Benito *et al*.^75^ recommendations, retaining only points separated by at least 0.04 decimal degrees (ca. 5 km; the spatial cell resolution). After this filtering process, a total of 720 presences were obtained (105 in native area, 315 in ASIA, 200 in USA-CA, 30 in AUS-NZ, and 70 in EUR, Fig. 1). These presences would thus be regarded as the updated distribution range of *L. lancifolium* at a global scale.

Regarding environmental variables, an initial set of 26 layers that may potentially influence *L. lancifolium* distribution was arranged. The initial set was composed by the following variables with a 2.5 arc-min resolution: 19 bioclimatic layers downloaded from the WorldClim website (https://www.worldclim.org), six topographical layers—including the elevation (downloaded from the WorldClim) and five derived layers (slope, aspect, compound topographic index, flow accumulation, and flow direction) calculated with the Spatial Analyst tool of ArcGIS version 10.2, and the index of anthropogenic impact called global human footprint^76^. To avoid model overfitting, a Pearson correlation analysis was performed between all possible combinations of the initial variables with data from all the different study areas. The selection of variables from groups of highly correlated (*r* ≥ |0.5|) ones was based on expert criteria of the species ecology and the biological significance of curve responses of presences and background points (Appendix B, Fig. S1 in Electronic Supplementary Material), the latter being interpreted as a sort of measure of use vs. availability of each resource. A variance inflation factor analysis (VIF) was also performed as a measure of multicollinearity^77^. The isothermality (bio3) was discarded for its high values of VIF (14.6) and its similarity in the curve response with respect to others (Appendix B, Fig. S1 in Electronic Supplementary Material). Human footprint was also rejected owing to many missing values of this variable in coastal presence points of the species. Only variables with VIF values < 5 were retained. After this selection process, we finally retained five climatic variables as data predictors: mean diurnal range (bio2), maximum temperature of the warmest month (bio5), mean temperature of the coldest quarter (bio11), annual precipitation (bio12), and precipitation seasonality (bio15).

### Niche comparison analyses on E-space

**Figure 2:**
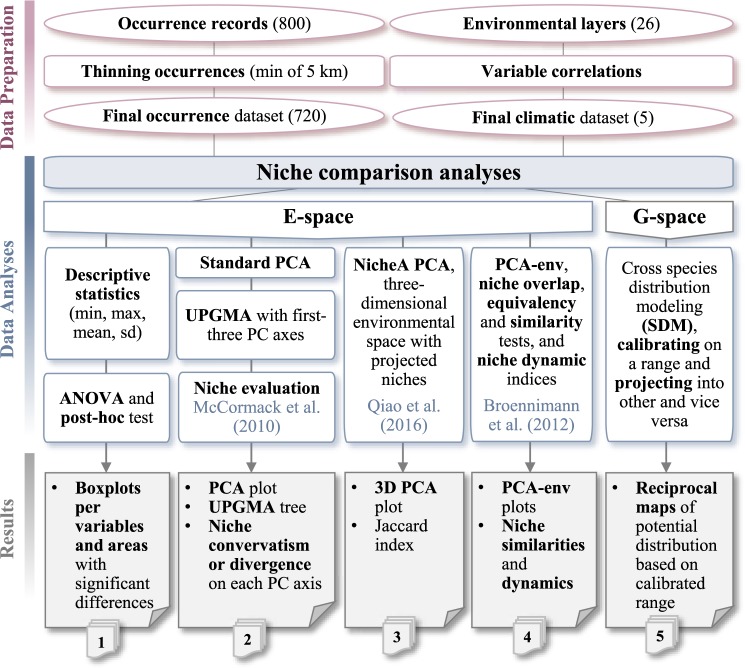
Workflow representation of raw data filtering, niche comparison analyses, and the resulting sets obtained with each methodological approach applied.

Niches of different areas of *L*. *lancifolium* were compared in both the environmental (E) and geographic (G) spaces, which have shown to complement each other in niche comparison studies^12,78^. We used different methodological approaches to assess niche conservatism or divergence among native and non-native ranges (see Fig. 2 for the workflow followed).

Firstly, descriptive statistics for each climatic variable were computed to outline the climatic data where the species inhabits in each of the ranges. To detect significant differences among ranges for each climatic variable, an analysis of variance was performed. Previously, the normal distribution of climatic data was checked with a Kolmogorov-Smirnov test (with the Lilliefors correction). As the variables did not fit the normality assumptions, a non-parametric pairwise Kruskal-Wallis test was performed using the Bonferroni correction for multiple pairwise comparisons with a post hoc test of Games-Howell.

Secondly, a principal components analysis (PCA; hereafter standard PCA) was conducted to examine the climatic variability of the realized niches in the occurrence ranges across the total climatic space. The values of the principal component (PC) axes were used to conduct a simple agglomerative hierarchical clustering method (UPGMA) in order to examine the relationships among realized niches of all areas. The distance matrix was calculated using the squared Euclidian distance employing the R function “dist” from the mean of the PCs with an eigenvalue ≥1. The resulting dendrogram was drawn with the average Hierarchical Clustering Method.

The approach described by McCormack *et al*.^6^ was used to assess possible niche shifts, in which observed niche divergence (*d*_n_) was tested against a null model of background climatic differences (background divergence, *d*_b_) in the axes of the PCA. Values for the five bioclimatic variables were sampled from all the occurrence points and from 1000 random background points extracted from a buffer influence zone of 20 km around presence points, using Buffer Tool implemented in ArcGIS. The five variables were reduced with the PCA with varimax rotation; only the PCs with an eigenvalue ≥1 were selected. Divergence on each niche axis (PC) was evaluated by comparing (by means of *t*-tests) the differences between the mean scores of the occurrence points for each of the two geographic areas that were compared (*d*_n_), and the differences between the 1000 background points (*d*_b_), with the null hypothesis being *d*_n_ = *d*_b_. Niche divergence is supported if *d*_n_ > *d*_b_ (and *d*_n_ is significant itself), whereas niche conservatism is supported if *d*_n_ < *d*_b_. Distributions of *d*_n_ and *d*_b_ were generated with 1000 bootstrap re-samplings, with the confidence interval for rejecting the null hypothesis set to 95%.

The recently developed software called NicheA^79^ was used to test the climatic niche overlap among the geographic areas studied. A multivariate and uncorrelated climatic space was built with the first three PCs from the PCA using all the values of the five selected climatic variables. The occurrences of different ranges were plotted in this climatic space in the form of minimum-volume ellipsoids (MVEs) and convex polyhedrons (CPs), which were generated around the occurrence points. In order to quantify niche similarities among regions, the Jaccard similarity coefficient^80^ was manually calculated through the overlap values for each paired comparison considering both the MVEs and CPs forms.

The methodological framework developed by Broennimann *et al*.^36^ was used to characterize native, archaeophyte, and neophyte niches through a PCA-env (a PCA calibrated on the entire environmental space of the study background). The backgrounds for each area were selected from a minimum convex polygon with a buffer size of 0.3 degrees, as recently proposed by Silva *et al*.^38^. Values of the five climatic variables were extracted from the backgrounds to construct the available environmental space represented by the two principal axes that account for the maximum variation of all the species’ ranges. We selected an environmental space with a resolution of 500 × 500 grid cells, each one representing a unique combination of climatic conditions. The original observed occurrences were corrected using a kernel function to smooth the distribution of densities and then were projected in the gridded environmental space. Finally, with the new dimensional surface, multiple range PCA-env plots were obtained representing all available climates and the occupied conditions simultaneously for the 20% and the 100% of occurrence density.

We further test the hypothesis of niche conservatism or divergence between realized niches of *L. lancifolium* in the five geographic areas using the comparative metric of niche overlap described as Schoener’s *D*^81,82^, a metric ranging from 0 (no overlap) to 1 (complete overlap). To evaluate whether two compared niches are more or less equivalent, or more or less similar than expected by chance, we performed the niche equivalency and niche similarity tests^36,82^, respectively. The first one evaluates whether niches are interchangeable only considering the climate space occupied by the exact occurrences, while the second, less conservative, accounts for the surrounding areas (background space) where the species occurs. Statistically, in a niche comparison 1 → 2, the occurrences from range 1 are randomly located on the same climatic space of the occurrences from 2 (in the equivalency test) or on the background of 2 (in the similarity test) for 100 times, thus obtaining simulated *D* values (*D*_sim_) that are compared to the real observed *D* ones (*D*_obs_). Three scenarios are possible when comparing both *D*s in a two-tailed test: (1) *D*_obs_ > *D*_sim_ (*p* < 0.05) means that compared niches (1 → 2) are more equivalent or more similar than expected by chance; (2) *D*_obs_ < *D*_sim_ (*p* < 0.05), when niches are less equivalent/similar than expected by chance; and (3) *D*_obs_ falls within 95% of *D*_sim_ values (*p* > 0.05), thus the hypothesis of retained niche equivalency or similarity cannot be rejected. We ran both analyses in a one-sided test, but each one was performed twice with options “greater” or “lower” (argument “alternative”, function “ecospat.niche.similarity.test”, R package “ecospat”^83^) to evaluate higher niche equivalencies/similarities than randomly expected and lower niche equivalencies/similarities than randomly expected, respectively.

Finally, to quantify the niche shifts, we calculated the three niche dynamic indices (stability, unfilling, and expansion^10,12^) based on the areas of the first two components of the PCA-env. Pairwise comparisons were carried out in both directions of distribution ranges (1 → 2 and 2 → 1). The values of all indices are expressed in percentage of gridded climate space occupied by total occurrence densities, ranging from 0 to 1 (values are considered relevant when > 0.1^84^). We used the total occurrence densities (i.e. the 100% of environmental space) considering Petitpierre *et al*.^12^ results, which revealed that niche metrics are not affected by using different percentiles to exclude marginal climates or kernel smoothing artefacts. Niche stability was employed as a measure of those climatic conditions that the species inhabits that are shared in the two ranges considered, by calculating the proportion of the total occurrence densities of range 1 that overlaps with range 2. Niche expansion describes new climate conditions occupied by the species in one of its ranges, also expressed by the proportion of total occurrence densities of range 1 that is not occupied in range 2. Niche unfilling classically refers to that climate available in invaded ranges but not yet occupied, indicating the level of climate equilibrium^84^, and was calculated as the proportion of total occurrence densities of range 2 that are not occupied in range 1.

The E-space analyses were conducted using SPSS version 22 (SPSS Statistics, Chicago, IL, USA) and R version 3.4.3^85^ in the RStudio platform^86^. For the analyses based on the PCA-env, we used the original R code reported in Broennimann *et al*.^36^ and later modified by Silva *et al*.^38^, which was run with slight modifications; these included the definition of regions independently by their own longitude and latitude limits, and the exportation of background polygons in ESRI shapefiles. The adapted R script applied here is included in Appendix C (Electronic Supplementary Files) and Rcode File.

### Niche comparison analyses on the G-space

For the analysis in G-space, we used the maximum entropy algorithm implemented in MaxEnt version 3.3^87^ to create reciprocal SDMs calibrated in the native area and projected into introduced ranges, and vice versa, and also calibrated in archaeophyte ranges and projected into neophyte ranges, and vice versa^23,31–33^. This approach allows identifying which geographic areas share similar climatic conditions between the calibrated and projected ranges. Additionally, results on potential distribution of the species in introduced locations could also be useful to infer the areas not currently occupied by the species but with suitable climatic conditions to prosper, thus directly indicating the magnitude and direction of possible future range expansions^7,8^.

For the reciprocal distribution models, we used the same uncorrelated five climatic variables as for the E-space (bio2, bio5, bio11, bio12, and bio15), which were clipped with ArcGIS to each of the five studied regions. All calibrated-projected modeling sets were performed applying the following conditions that have shown robust results in former studies^88^: 20 replicates using the bootstrap method as resampling strategy, selecting 25% of random occurrence records for model testing, and with options extrapolate, do clamping, and fade by clamping selected. Model accuracy and its predictive capacity were evaluated using the area under curve (AUC^89^), indicating 0.7 to 0.9 good model fitting and values above 0.9 excellent performance. The resulting continuous output maps were modified and exported with ArcGIS considering as cut-off value “the maximum sensitivity plus specificity logistic threshold” as recommended by Liu *et al*.^90^; such a threshold has been widely used^88,91,92^. At the end, following Gallardo *et al*.^93^ approach, the threshold-delimited maps were used to calculate the percentage of the total occurrences in the model projected area that were located inside suitable conditions (e.g. the percentage of archaeophyte occurrences correctly predicted by the model calibrated in the native range and vice versa).

## Results

### Niche variation on E-space

**Figure 3:**
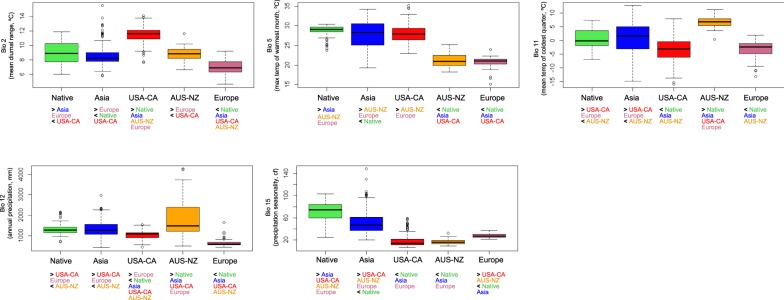
Boxplots of five climatic variables selected for the native (Korea), archaeophyte (ASIA), and neophyte (USA-CA, AUS-NZ, EUR) ranges of *Lilium lancifolium*. Significant differences (*p* < 0.05) for the climatic variables between regions are indicated by > or < symbols below each plot (see Appendix B, Table S2 in Electronic Supplementary Material for details).

The analysis of variance of climatic variables showed that bio15 (precipitation seasonality) was significantly different for all native vs. introduced comparisons, specifically being higher in native range (Fig. 3; Appendix B, Table S2 in Electronic Supplementary Material). For the climatic niche comparison between native vs. archaeophyte (ASIA), three variables presented significant differences (*p* < 0.05; bio2, mean diurnal range; bio5, maximum temperature of the warmest month; bio15) whereas the other two (bio11, mean temperature of the coldest quarter; bio12, annual precipitation) resulted statistically indistinguishable (*p* > 0.05). Native and EUR neophyte range showed statistically differences in climatic values for all the variables. The other two neophyte ranges (USA-CA and AUS-NZ) also turned out to be significantly different from native, except for bio5 in the native vs. USA-CA comparison, and for bio2 in the native vs. AUS-NZ comparison (Fig. 3; Appendix B, Table S2 in Electronic Supplementary Material).

**Figure 4:**
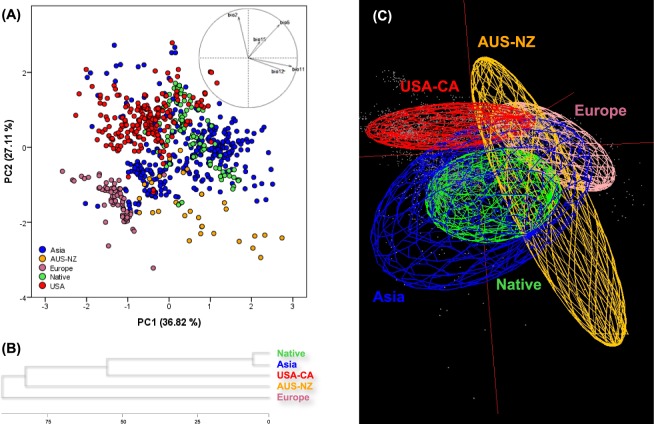
(**A**) Standard Principal Component Analysis (PCA) performed with climatic values of *Lilium lancifolium* for the occurrences of the native, archaeophyte (ASIA), and neophyte (USA-CA, AUS-NZ, and EUR) ranges. (**B**) UPGMA clustering representation derived from the coordinates of occurrences on the first three axes of the standard PCA. (**C**) Minimum-volume ellipsoids (MVE) of different regions into the environmental space performed with NicheA.

In the standard PCA analysis (Fig. 4A), the first three PCs accounted for the 84.7% of the total climatic variance (PC1 = 36.8%, PC2 = 27.1% and PC3 = 20.8%; Appendix B, Table S3 in Electronic Supplementary Material). The first component (PC1) was mainly explained by bio11 and bio12, the second (PC2) by bio2 and bio5, and finally the third (PC3) by bio15 (Appendix B, Table S3 in Electronic Supplementary Material). The distribution of native occurrences in the climatic PCA space overlapped mainly with ASIA and USA-CA ranges, in contrast to AUS-NZ and EUR, which appeared slightly distant (Fig. 4A). ASIA was the species range that overlapped most with the other regions, and also was the most widely distributed. The UPGMA dendrogram (Fig. 4B), derived from the PC values of the standard PCA analysis, showed that ASIA is the range most closely related to the native area, whereas EUR was the climatically most distant range from the native area.

Values of the Jaccard similarity index were generally low (Table 1). Only between ASIA and native ranges the overlap values measured with both forms (MVE and CP) were significantly similar; it should be noted that the native form was inside the ASIA form, thus the overlap volume was equal to the native form volume (Fig. 4C and Appendix B, Fig. S3 in Electronic Supplementary Material). The forms for AUS-NZ and EUR ranges overlapped with all forms for the other ranges (although the overlap between EUR and native ranges was almost negligible). In contrast, the form for USA-CA did not overlap with that for native (Jaccard index = 0), but partially with that for ASIA range (Jaccard index > 0).

**Table 1:** Jaccard index comparisons of similarity among ranges of *Lilium lancifolium*.

	Native	ASIA	USA-CA	AUS-NZ	EUR
Native	—	**0.21**/**0.19**	0/0	**0.09**/0.02	0.01/0
ASIA		—	**0.04**/0	**0.09**/0.02	**0.07**/0.01
USA-CA			—	**0.06**/0.02	**0.04**/0
AUS-NZ				—	**0.09**/0
EUR					—

The first value corresponds to the Jaccard index measured on minimum-volume ellipsoids (MVE), while the second one on convex polyhedron (CP), representing both the environmental conditions of each region. In bold, Jaccard indices > 0.02.

Regarding the McCormack *et al*.^6^ analysis, results did not clearly point to a complete niche conservatism (*d*_n_ < *d*_b_) or divergence (*d*_n_ > *d*_b_, and *d*_n_ is significant) scenario for any of the comparisons. For almost all comparisons, either conservatism or divergence were proposed for alternative PC axes (Appendix B, Table S4 in Electronic Supplementary Material), likely indicating that the pattern observed was highly dependent on the most contributing variables to the PC axis considered. The comparison native range vs. EUR was the only case in which results for the three PCs were not conclusive of niche divergence or conservatism.

**Figure 5:**
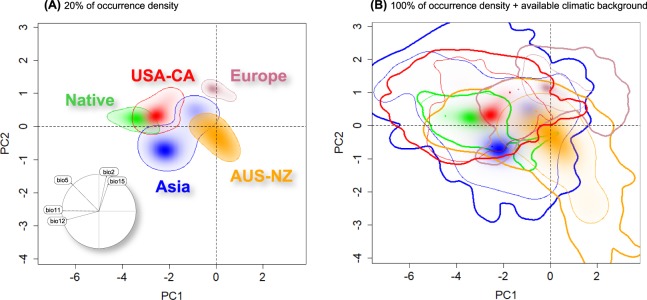
Global climatic space constructed over all background areas and realized niches of *Lilium lancifolium*, showing overlaps between native (Korea), archaeophyte (ASIA) and neophyte (USA-CA, AUS-NZ, EUR) distribution ranges, plotting a solid line representing the 20% of occurrence density (**A**), and 100% of occurrence density with a thin line and 100% of available climatic background with a thick line. (**B**) The left graph includes the contribution and direction of each variable to the two-first components of the PCA-env.

In the PCA-env analysis (Fig. 5), the first two axes explained 64.1% of the total variation of climatic conditions for the *L. lancifolium* ranges (PC1 = 37.1% and PC2 = 27.0%). The first component (PC1) was mainly explained by bio 11, whereas the second was principally loaded by bio12 (Appendix B, Fig. S2 in Electronic Supplementary Material). The multiple niche plot displaying the 20% of occurrence density (Fig. 5A) showed a close relation of the realized niche of the native area with that of USA-CA, and to a lesser extent with ASIA. Conversely, AUS-NZ and EUR placed clearly distant from the native shadow. When the 100% of occurrence density was plotted in the PCA-env space, a high overlap in climatic space was detected among ranges (Fig. 5B). Specifically, the ASIA range showed the highest niche breadth, encompassing almost all climatic niche space of native and USA-CA regions, and about half of AUS-NZ and EUR ranges. In the two multiple niche PCA-env plots (Fig. 5A,B), the EUR range of *L. lancifolium* showed the smallest niche breadth and was the most climatically differentiated compared to the other ranges.

The overlap *D* values between compared areas were generally low (Table 2), the native and USA-CA being the most similar ranges in their realized niches (*D* = 0.27), whereas the native and EUR ranges were completely differentiated (*D* = 0.00). The climatic niche occupied by the archaeophyte range (ASIA) presented the highest overlap values when it was compared with the other four ranges (*D* = 0.10–0.24). In the niche equivalency test (in which only climatic space occupied by the occurrence records are considered), we found that only the niche from the archaeophyte range (ASIA) resulted interchangeable with the neophyte ranges (USA-CA, AUS-NZ, and EUR); in contrast, the archaeophyte (ASIA) and the neophyte ranges of AUS-NZ and EUR were not equivalent to the native area (Table 2). In the niche similarity test (background areas are also considered), the native niche was more similar than expected by chance to both the archaeophyte (ASIA) and the USA-CA neophyte range. Interestingly, the ASIA range shows clear signals of niche conservatism when compared with all other ranges (the native and the three neophyte areas). Not one similarity test indicated niche divergence between compared ranges (Table 2).

**Table 2:** Metrics of niche dynamics comparing pairs of *Lilium lancifolium* ranges (1 → 2), following the methodological framework developed by Broennimann *et al*.^36^.

*Lilium lancifolium* distribution ranges		Niche Overlap (*D*)	Equivalency test (*p*-value)		Similarity test (*p*-value)		Niche unfilling	Niche stability	Niche expansion
1	2		less eq. (DIV)	more eq. (CON)	less sim. (DIV)	more sim.(CO N)			
Native	ASIA	0.10	**0.01**	1.00	1.00	**0.01**	0.62	1.00	0.00
	USA-CA	0.27	0.59	0.55	0.83	**0.01**	0.32	0.89	0.11
	AUS-NZ	0.09	**0.01**	1.00	1.00	0.24	0.91	0.14	0.86
	EUR	0.00	**0.01**	1.00	0.79	1.00	1.00	0.00	1.00
ASIA	Native	0.10	**0.01**	1.00	1.00	**0.01**	0.00	0.38	0.62
	USA-CA	0.19	1.00	**0.01**	1.00	**0.01**	0.01	0.61	0.39
	AUS-NZ	0.24	1.00	**0.01**	1.00	**0.01**	0.35	0.40	0.60
	EUR	0.14	1.00	**0.01**	0.99	**0.05**	0.12	0.06	0.94
USA-CA	Native	0.27	0.61	0.53	1.00	**0.01**	0.11	0.68	0.32
	ASIA	0.19	1.00	**0.01**	0.99	**0.03**	0.39	0.99	0.01
	AUS-NZ	0.07	0.64	0.40	0.93	0.08	0.75	0.18	0.82
	EUR	0.02	**0.01**	1.00	0.76	0.21	0.96	0.01	0.99
AUS-NZ	Native	0.09	**0.01**	1.00	0.77	0.22	0.86	0.10	0.90
	ASIA	0.24	1.00	**0.01**	1.00	**0.01**	0.60	0.65	0.35
	USA-CA	0.07	0.74	0.23	0.89	0.12	0.82	0.25	0.75
	EUR	0.03	**0.05**	0.99	0.82	0.26	0.81	0.05	0.95
EUR	Native	0.00	**0.01**	1.00	0.68	1.00	1.00	0.00	1.00
	ASIA	0.14	1.00	**0.01**	0.97	**0.02**	0.94	0.88	0.12
	USA-CA	0.02	**0.01**	1.00	0.78	0.28	1.00	0.04	0.96
	AUS-NZ	0.03	**0.05**	0.99	0.81	0.28	0.95	0.19	0.81

Interpretation of niche parameters: overlap (*D*; 0 = no overlap, 1 = complete overlap), less equivalent or less similar (significant values when *p* < 0.05, indicating that niches are less equivalent/similar than expected by chance), more equivalent or more similar (significant values when *p* < 0.05, indicating that niches are more equivalent/similar than expected by chance), unfilling (0 = totally filled, 1 = totally unfilled), stability (0 = not stabilized, 1 = totally stabilized), expansion (0 = in equilibrium, 1 = fully in expansion). Note that the last three metrics were calculated by the intersection between the thin lines of compared ranges (100% percentile of occurrence density; Fig. 5B). Abbreviations used: eq. = equivalent; sim. = similar; DIV = divergent; CON = conserved.

On the first temporal introduction stage (i.e. the spread from the native to the archaeophyte areas), the species expanded its niche in a 0.62 proportion, and completely filled the primary niche space found in native range (Table 2). On the second colonization period (spread to neophyte areas), the species resulted in a moderate niche expansion respect to the native range in the case of USA-CA (0.32) but in a very high expansion in AUS-NZ and EUR, with values of 0.90 and 1.00, respectively. The area with greater amount of suitable habitat but not yet occupied was EUR (unfilling = 0.94–1.00), followed by AUS-NZ (unfilling = 0.60–0.86), and to a lesser extent by USA-CA (unfilling = 0.11–0.96), as revealed by the unfilling values in relation to other ranges and background availability (Fig. 5B; Table 2). On the contrary, the species in its native area is already established in almost all its potential climate (Fig. 5B). Finally, in ASIA the species could explore some small new climatic areas currently occupied in AUS-NZ and EUR (Fig. 5B; Table 2).

### Reciprocal distribution models on G-space

**Figure 6:**
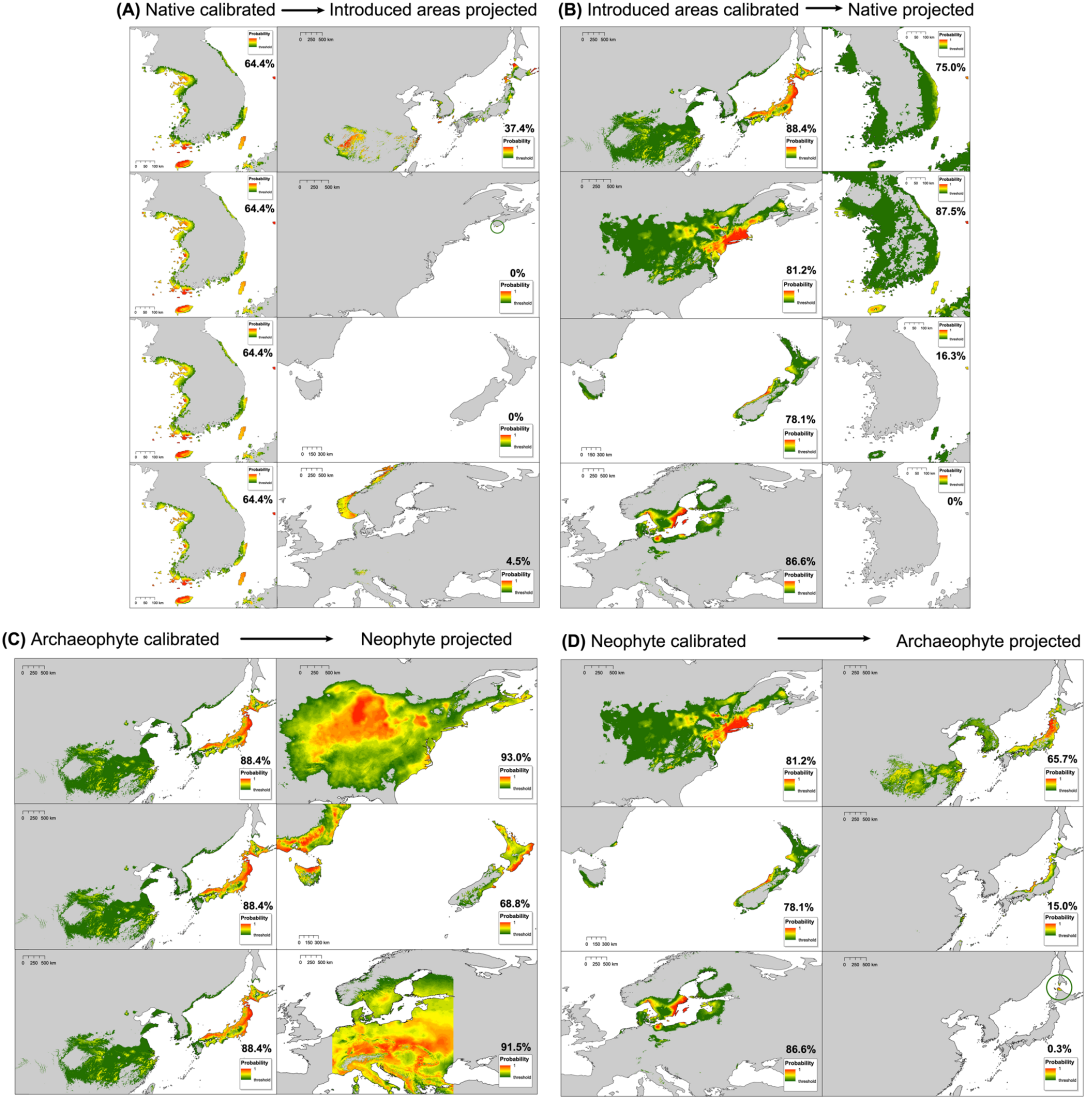
Reciprocal species distribution models (SMDs) calibrated (**A**) on the native range with its occurrence records and climatic data, and projected into climatic background conditions of introduced ranges (N → I), (**B**) on the introduced ranges and projected to the native area (I → N), (**C**) on the archaeophyte range (ASIA) and projected to neophyte ranges (USA-CA, AUS-NZ, and EUR) (Arc → Neo), and (**D**) on the neophyte ranges projected to the archaeophyte range (Neo → Arc). The percentage of correctly predicted occurrences by each model is also indicated. Color ramp represents habitat suitability measured in probability of occurrence, from green (showing low suitability) to red (high suitability). Geographic areas with probability values below selected threshold are colored in gray.

As a general pattern, all reciprocal distribution models resulted in moderately high model performance and prediction power (average AUC = 0.886 ± 0.042). The highest transferability in native calibrated vs. introduced projected (Fig. 6A) was detected between native and the archaeophyte ranges, while very low matching of climate space was found between the native model and all neophyte areas (USA-CA, AUS-NZ, and EUR). For the models built with native data, only a 64.4% of present occurrences were located in suitable areas (Fig. 6A on left). The models performed in introduced ranges and projected to the native range (Fig. 6B) showed both low (in AUS-NZ and EUR) and high (ASIA and USA-CA) predictive power for native occurrences. High percentages of correctly predicted occurrences were detected in archaeophyte built models projected to neophyte ranges (Fig. 6C). In contrast, for neophyte to archaeophyte projections, only the USA-CA model predicted a high proportion of ASIA occurrences (Fig. 6D).

## Discussion

### Niche characteristics in *Lilium lancifolium*: from native to archaeophyte, and from archaeophyte to neophyte ranges

The climatic niche assessment of *L. lancifolium* has helped to shed light on our understanding of how climatic niches of non-native plants can be transformed in a large spatial (along continents) and temporal (hundreds of years) framework. To our knowledge, the case presented here is the first niche characterization study of a native plant species with two hypothesized introduction surges, one well before AD 1500 (i.e. behaving as an archaeophyte), and the other after AD 1500 (i.e. as a neophyte). The methodological exploration with the most commonly used (but also cutting-edge) approaches for niche comparisons (Fig. 2), both in E and G spaces, allowed us to draw some conclusions.

Firstly, it should be noted that *L. lancifolium* in its native range seems to be occupying a rather small climatic space, especially when compared with the non-native areas (Figs 3–5). In the first invasion step, when the species was introduced from the native to its non-native purportedly archaeophyte area (East Asia), niche comparison analyses reveal that *L. lancifolium* would have completely occupied the ancestral niche and, additionally, its climatic niche would have increased by 62% with respect to the native one (Table 2). This geographic range expansion from a local to a regional scale could have promoted the occupancy of novel and previously non-existing native climatic niches on the Korean Peninsula, which indicates the wider ecophysiological tolerance of *L. lancifolium* outside its native area. In the second introduction event of the species to other continents (that is, the areas tentatively regarded as neophyte ranges), the high niche affinity with archaeophyte rather than with native climate lead us to hypothesize that a propagule reservoir from the archaeophyte range (China or Japan) probably spread into neophyte ranges (USA-CA, AUS-NZ, and EUR). As shown by the equivalency and similarity tests (Table 2), in general there are closer climatic similarities between archaeophyte and neophyte ranges than between native and neophyte ranges (with the exception of USA-CA). Furthermore, the accurate prediction of the neophyte occurrences with the archaeophyte calibrated models (Fig. 6C) may suggest that the species could have survived and established itself in neophyte ranges due to its preadapted conditions and its matching pattern of suitable climates. Many studies of plant invasion have documented in detail the effect of a previous preadaptation condition of the imported pools, which confers a clear ecological advantage, especially when climates between source and introduced regions are fully or partially analogous (climate match hypothesis^7,16,94^).

In addition to the climate similarity premise, the knowledge of the historical commercial routes also gives support to the proposed colonization history scenario. Although the species might have firstly reached Europe from Japan at the end of 17^th^ century thanks to the Dutch East India Company (after the Shimabara Rebellion of 1637 the Dutch were the only of the western powers that were allowed to commerce with Japan^95^), it seems that the species did not “jump” into western gardens until its documented introduction from Guangzhou (Canton) in China in the early 19^th^ century^50,53^. Other contemporary introduction events from either China or Japan are unknown, although they are plausible: Chinese trade with the west via the Guangzhou port has been very active since the middle 18^th^ century, whereas commercial exchanges with Japanese ports were also common, first through the Dutch, and significantly increasing with the Perry Expedition and Meiji Restoration by the middle 19^th^ century^96^. According to Ohkawa^97^, *L. lancifolium* was among the three lilies most exported from Japanese ports in the 1870s, and it seems that arrivals of the plant to Europe from Japan were common during the 1860s and 1870s^98^. An introduction from Korea is, in contrast, very unlikely, as the peninsula remained completely isolated from the outside world until the late 19^th^ century (during this century, Korea was generally regarded as “The Hermit Kingdom”^99^). Apart from climatic and historical data evidences, to further shed light on the species colonization routes, a population genetic study (using high-resolution DNA markers) would be needed^100^.

Overall, niche conservatism with an additional niche expansion is proposed for the transition of *L. lancifolium* from its native to its archaeophyte range. For the archaeophyte to neophyte transition, although some results may suggest a non-evolutionary niche shift, the species is partially filling marginal or narrow climates from the whole archaeophyte niche (e.g.^101^), thus a conservatism scenario would be more appropriate. It should be noted that we did not find quantifiable evidence across all analyses showing that niches have completely differentiated between native and neophyte ranges. The relative lack of analogue climates (i.e. non-overlap background conditions) between native and neophyte ranges in *L. lancifolium* (especially for AUS-NZ and EUR) would be interpreted as a true niche shift. However, conclusions of niche divergence in non-analogue climatic space would not be well-founded, as these can simply reflect the lack of adequate climates in the introduced range^38^ and, thus, they would not be the consequence of acting ecological processes^11^. A true niche shift can only be interpreted when the background is identical between native and invaded areas and there is a selection of different environments by the species^18^.

The fact that the climatic niche of *L. lancifolium* has not significantly diverged along continents and hundreds of years could be related to its mode of reproduction and cytotype structure. Even though it is well known that in its native range two cytotypes are currently coexisting (diploids and triploids, with the former exclusively occurring on coastal areas and the latter mainly distributed in inland regions^39–43^), such detailed information on cytotype composition and distribution is, unfortunately, not available for the species in the introduced areas. It is assumed, however, that in the introduced areas *L. lancifolium* occurs as triploid. Almost all the available chromosome counts support this assumption, including numerous ones from wild populations of China and Japan, and also a few from cultivated populations of Romania and both wild and cultivated populations from the United States (Appendix B, Table S1 in Electronic Supplementary Material). It should be noted here that the plants brought to England in 1804 from China were most likely triploid, judging from comments of Chandler *et al*.^102^ in their paper reporting a triploid chromosome count for the species: “the type clone of the triploid *Lilium tigrinum* […] was first sent from China to the Royal Botanic Gardens at Kew, England, by William Kerr in 1804 and propagated since solely by asexual means for horticultural culture”. Therefore, if the triploid has been the predominant cytotype in the spread of the species outside its native area, the low niche divergence may be simply explained by the vegetative reproduction of the triploids, a factor that is hindering the activation of natural selection and the generation of new genetic diversity. In some extreme cases, triploid cytotypes can be limited to a single clone that is sometimes spread over hundreds of kilometers (e.g.^103,104^) and, in fact, they can function as individuals with extremely long life-spans (e.g. *Lomatia tasmanica*^105^; see also^106^ for a review). Indeed, Chung *et al*.^42^ found that, within the native range, triploids showed much lower levels of genetic polymorphism compared to diploids, with only six clones detected throughout Korea. Recent studies (e.g.^27^) suggest that invasive species prosper in the new environments thanks to selection acting on the existing genetic variation rather than producing new mutations (see^4^ or^107^ for a theoretical framework). The much lower genetic diversity of the triploids in the native range compared to the diploids (percentage of polymorphic allozyme loci, %*P* = 16.1 vs. 55.8; mean number of alleles per locus, *A* = 1.16 vs. 1.59^42^) would have effectively limited the capacity of adaptation of colonizing propagules and hindered the ability for shifting its niche^108,109^. The admixture of genetically divergent lineages may also promote niche shifts (as observed for example in *Schinus terebinthifolius*^110^). In *L. lancifolium*, even in the case of multiple introductions, the triploid clones are genetically very close (just differing in a few alleles and, at the same time, being allele subsets of the diploid populations^42^), thus limiting any possibility of niche shifts. However, recent studies^111^ suggest that phenotypic plasticity may allow plants to colonize new environments even when genetic diversity is low; studies focused on phenotypic plasticity would be very useful, thus, to clarify the role of genetic diversity in the colonization strategy of *L. lancifolium*.

An extensive cytogenetic study to explore the distribution of cytotypes of *L. lancifolium* across all invasion areas would help to shed light on the effects of cytotype composition on the observed patterns of niche dynamics. Such a study would also help to explain the inconsistences detected in the reciprocal SDM models; for example, in spite of the high niche affinity of archaeophyte–native areas (Fig. 4), the reciprocal SDM models showed relatively low prediction values when models were calibrated in the native and projected to the archaeophyte range (only 37.4% were correctly predicted; Fig. 6A). A straightforward and reasonable interpretation of this pattern would be a niche expansion of the species in the archaeophyte range. An alternative interpretation might be, however, that there is some degree of niche divergence between diploids and triploids in the native range that would remain undetected in the previous study of Chung *et al*.^42^. In such a case, the native models would have some inherent limitations, as exemplified in the poor ability to estimate the current known occurrence records of *L. lancifolium* (only 64.4% of occurrences were predicted as suitable in its native range; Fig. 6A). The low predictive ability of the native model could be due to the fact that most occurrences of the species in Korea are from coastal areas (all the diploid occurrences plus almost half of the triploid ones^42^), and, thus, inland areas where the triploid is mainly found do not appear as suitable. According to Chung *et al*.^42^, the low occurrence of triploids on western and southern coastal areas of the Korean Peninsula may be due to the fact that most of these niches were already occupied by the diploids at the time of triploid formation (i.e. competition between cytotypes).

### Invasion stages in archaeophyte and neophyte ranges

The initial expectations on detecting signals of an advanced and an earlier process of colonization in the archaeophyte and neophyte ranges, respectively, have been confirmed here with the niche analyses performed. On the one hand, in the archaeophyte range it seems that the relatively long time period since the first arrival of *L. lancifolium* (about two millennia^51,53^) would have allowed the total filling of the native climatic niche and, additionally, the capacity to adapt and prosper in other new climatic regions. The present-day snapshot of the ratio between realized vs. available climatic background in the archaeophyte range (the comparison between thin and thick lines in Fig. 5B) indicates that the species is occupying a significant fraction of potential habitats in this region, with the not yet occupied zones probably being unsuitable due to various other abiotic or biotic constrictions. For example, competition with truly native *Lilium* species is likely, especially for the phylogenetically most closely related congeners (as these tend to conserve the same niche, i.e. “phylogenetic inertia”^112^). Certainly, the extended time elapsed since the species was introduced in the non-native areas of East Asia could have played an important role in the currently observed advanced and fairly stabilized invasion stage. However, other factors, including several introduction events, mixed introduced propagules, and large propagule number (e.g.^113–116^), or even the probability of later exchanges of propagules among introduced regions within the archaeophyte range, would have accelerated niche stabilization and filling of the ancestral habitat conditions. This is in agreement with the idea that the species range expansion along its archaeophyte area seems closely related to a human-mediated interaction as proposed for dispersal pathways of cultivated species (see^117^), rather than an exceptional dispersion capacity of bulbils, considering the wide and continued commercial and medicinal use of the species in East Asia (see Introduction for details). In this case, the niche stabilization could have been favored by a temporal component, but also enhanced and accelerated due to the anthropogenic factor.

On the other hand, in the neophyte areas the much shorter time period since the initial colonization (about two hundred years) could have contributed to the only partial filling of the available archaeophyte climatic niche conditions. As proposed above, the species could probably have arrived to neophyte ranges through a subset of archaeophyte propagules adapted to local climatic conditions. In addition, the species seems to prosper well or to actively select those habitats with similar climates to the archaeophyte range from all its neophyte available background. In this case, a founder effect scenario derived from a bottleneck event, or a colonization time lag effect^15,23,118,119^ could be suggested as potential driver mechanism, such as previously proposed for other species with a partial filling of source climates^12,24,101^. Indeed, genetic data would be desirable to explore both proposed hypotheses in more depth. Other non-time related factors may be also limiting the species expansion in neophyte ranges, including environmental requirements for the species (mycorrhizae or adequate soil conditions^120,121^), physical barriers to dispersion (mountain chains; e.g.^24,122^), biological interaction constraints^123^, or restricted human-mediated dispersal (low exchanges for gardening purposes). As a high proportion of unoccupied but suitable climatically available habitat was detected in the realized neophyte ranges (Figs 5B and 6), it seems plausible that the species may be currently limited by dispersal capabilities or life-history constraints^124^, rather than climatic or physiological tolerance limits^125^. Among the three neophyte areas of *L. lancifolium*, the lowest rate of niche unfilling corresponds to USA-CA (0.11 vs. 0.86 and 1.00 for AUS-NZ and EUR, respectively; Table 2). Such results are not surprising, given that there is a much lower tradition to cultivate *L. lancifolium* in Europe or in Australia/New Zealand compared to North America, which explains why the species is much less frequent in the wild in the former areas. Indeed, the first documented wild occurrence in Australia/New Zealand is very recent (from the late 1950s^64^), as for Europe^126^. In contrast, in North America the species is a well-known and extremely popular ornamental plant, with a long tradition of cultivation in backyards (e.g.^127–129^) a factor that with no shadow of doubt would have favored its human-mediated transport and dispersal. This fact would have additional conservation or management implications, since the establishment area of *L. lancifolium* in its non-native areas could directly depend on a human-mediated interaction (the triploid cytotypes are sterile), as proposed at a global scale in cases of cultivated introduced species^11^.

The pattern observed for *L. lancifolium* suggests that a long period of time since the first arrival of a non-native species could contribute to the detection of: (1) a high niche stabilization with the receiver environment and community (e.g. in the archaeophyte range almost all habitats with available and suitable climates are occupied, while in the neophyte ranges there is still a high proportion of climates to be filled); and (2) niche conservatism between source and introduced areas (e.g. native and archaeophyte ranges are more similar to each other than archaeophyte and neophyte ranges). This study also highlights the idea that niche similarities could be eroded at each colonization step, like as a “stepping stone” scheme, from the native to archaeophyte step, and from the archaeophyte to neophyte step, the most divergent niches thus being those from native to neophyte ranges. As previously reported for other plant groups^11,130^, intercontinental range expansions might also facilitate niche divergence, while more regional expansions might be related to a niche conservatism scenario.

## Supplementary information

Supplementary Material
