# Supplementary material for: Climatic niche characteristics of native and invasive Lilium lancifolium
Source: Sci Rep. 2019 Oct 4;9:14334. doi: 10.1038/s41598-019-50762-4 (PMC6778149; doi:10.1038/s41598-019-50762-4)
Supplement: Supplementary file 1 — Supplementary Material [file 41598_2019_50762_MOESM1_ESM.docx]

**Supplementary Material:**

**Climatic niche characteristics of native and invasive *Lilium lancifolium***

**Sonia Herrando-Moraira^1^, Neus Nualart^1^, Albert Herrando-Moraira^2^, Mi Yoon Chung^3^, Myong Gi Chung^4^ & Jordi López-Pujol^1^**

^1^Botanic Institute of Barcelona (IBB, CSIC-ICUB), Barcelona ES 08038, Catalonia, Spain. ^2^Polytechnic University of Catalonia, Terrassa ES 08222, Catalonia, Spain. ^3^Research Institute of Natural Science (RINS), Gyeongsang National University, Jinju 52828, Republic of Korea. ^4^Division of Life Science and RINS, Gyeongsang National University, Jinju 52828, Republic of Korea

Correspondence and requests for materials should be addressed to M.G.C. (email: [mgchung@gnu.ac.kr](mailto:mgchung@gnu.ac.kr)) or J.L.-P. (email: [jlopez@ibb.csic.es](mailto:jlopez@ibb.csic.es))

S.H.-M. and N.N. contributed equally to the work.

**Appendix A: References holding *Lilium lancifolium* occurrences**

**Web portals with *Lilium lancifolium* occurrences**

- Alabama Plant Atlas (<http://www.floraofalabama.org/>)
- Atlas of Living Australia (<http://biocache.ala.org.au>)
- Biodiversity of the Hengduan Mountains (<http://hengduan.huh.harvard.edu/fieldnotes>)
- BisQue database (<https://bisque.cyverse.org/>)
- Brooklyn Botanic Garden’s Herbarium (<http://herbarium.bbg.org/ailanthus/search.php> )
- Chinese Virtual Herbarium (<http://www.cvh.ac.cn>)
- Connell Memorial Herbarium (<http://unbherbarium.ca/>)
- Consortium of Midwest Herbaria (<http://midwestherbaria.org/portal/>)
- Flick (<https://www.flickr.com> )
- Flickriver (<http://www.flickriver.com>)
- Flora of Hokkaido (<http://www.hinoma.com/maps/>)
- Flora of Pan-Himalaya (<http://www.flph.org/>)
- GBIF, Global Biodiversity Information Facility (<http://www.gbif.org>)
- GISIN, Global Invasive Species Information Network (<http://www.gisin.org/>)
- Great Plains Regional Herbarium Network (<http://ngpherbaria.org/portal/index.php>)
- Harvard University Herbaria & Libraries (<https://huh.harvard.edu/>)
- iNaturalist.org (<https://www.inaturalist.org>)
- Intermountain Regional Herbarium Network (<http://www.intermountainbiota.org>)
- Iowa Plants (<http://iowaplants.com/>)
- Kasviatlas (<http://koivu.luomus.fi/kasviatlas/>)
- Minnesota Plants (<http://minnesotaseasons.com/Main/Plants.html>)
- Minnesota Wildflowers (<https://www.minnesotawildflowers.info>)
- NatureWatch NZ (<http://naturewatch.org.nz> )
- Online Virtual Flora of Wisconsin ([http://wisflora.herbarium.wisc.edu](http://wisflora.herbarium.wisc.edu/))
- Plant Photo Bank of China (<http://www.plantphoto.cn>)
- Plants Index (<http://www.plantsindex.com/>)
- Project Noah (<http://www.projectnoah.org>)
- SEINet, Arizona and New Mexico Chapter (<http://swbiodiversity.org/seinet/>)
- SERNEC, SouthEast Regional Network of Expertise & Collections (<http://sernecportal.org> )
- Taiwan Biodiversity Information Facility (<http://taibif.tw/>)
- The Australasian Virtual Herbarium (<https://avh.chah.org.au/>)
- The Herbarium at AUNE (<http://www.auneherbarium.org/>)
- The New York Botanical Garden Virtual Herbarium (<http://sciweb.nybg.org/science2/vii2.asp>)
- Tokushima Prefectural Museum (<http://www.museum.tokushima-ec.ed.jp/database.htm>)
- Tompkins County Flora Project (<http://herbarium.bh.cornell.edu/>)
- Tropicos (<http://www.tropicos.org>)
- University of Florida Herbarium (<https://www.floridamuseum.ufl.edu/herbarium/>)
- University of Maine Herbaria (<http://herbaria.umaine.edu/index.php>)
- University of Massachusetts Herbarium (<https://www.bio.umass.edu/biology/facilities/herbarium>)
- Weeds of Australia (<http://keyserver.lucidcentral.org/weeds/data/media/Html/>)

**Papers or books with *Lilium lancifolium* occurrences**

Belyanina Y, Denisova Y (2016) The analysis of a specific variety of the rare and protected plants and the animal especially protected natural territories of southeast part of the island of Sakhalin. Estestv Mat Nauk Sovrem Mire (Nat Mat Sci Mod World) 2:45–52 (in Russian)

Blake S (1914) Six weeks' botanizing in Vermont— III. Notes on the plants of Swanton and vicinity. Rhodora 16:38–41

Choi YE, Kim CH (2013) A Study on characteristics of the endangered species *Psilotum nudum* habitat in Jeju Island*.* J Korea Soc Environ Restor Technol 16:1–17 (in Korean)

Chung MY, López-Pujol J, Chung JM, Kim KJ, Park SJ, Chung MG (2015) Polyploidy in *Lilium lancifolium*: evidence of autotriploidy and no niche divergence between diploid and triploid cytotypes in their native ranges. Flora 213:57–68

Cui J, Yang X, Zhang K, Jia Y (2016) Phylogenetic relationship of lilies (*Lilium*) analyzed based on trnH-psbA barcode technology, Mol Plant Breed 7(27):1–9

Du Y-P, Wei C, Wang Z-X, Li S, He H-B, Jia G-X (2014) *Lilium* spp. pollen in China (Liliaceae): Taxonomic and phylogenetic implications and pollen evolution related to environmental conditions. PLoS ONE 9:e87841

Fogg J (1930) The flora of the Elizabeth Islands, Massachusetts. Contr Gray Herb 91:119–281

Fukuoka N, Kurosaki N, Takahashi A (2007) Vascular plants of Hyogo Prefecture 9. Hum Nat 18:85–117 (in Japanese)

Greaves JM (1982) New records of vascular flora from Gloucester County, Virginia. Va J Sci 33:47–63

Green SA, Hill ES (1914) Facts relating to the history of Groton, Massachusetts, vol 2. J. Wilson and Son, Cambridge.

Greller AM, Lotowycz GE, Moore G et al (2005) Vascular flora of Caumsett State Historic Park, Lloyd Neck, Long Island, New York, with notes on the vegetation. J Torrey Bot Soc 132:149–168

Griffin D, Doug K, MacQuerrie I (1988) Wild in the city: Urban botany. Isl Mag 23:16–18

Gu X, Zhang Y-L, Niu L-X (2013) Pollen morphology observation of 15 wild lilies from four provinces in Western China. Acta Hortic Sinica 40:1389–1398 (in Chinese)

Healy AJ (1958) Contributions to a knowledge of the adventive flora of New Zealand, No 6. T Roy Soc NZ 85:531–549

Hirose T, Ota T, Nakagawa H, Obata K, Sakurai T, Takano N (2001) The vascular plant flora of the secondary forest in Iwai. Bull Ibaraki Nat Mus 4:131–144 (in Japanese)

Hwang HS, Yang JC, Oh SH, Lee YM, Chang KS (2013) A study on the flora of 15 islands in the Western Sea of Jeollanamdo Province, Korea. J Asia Pac Biodivers 6:281–310

Igarashi H (2013) Checklist of naturalized plants from Rishiri Island, Rebun Island, Teuri Island, Yagishiri Island and Okushiri Island. Rishiri Stud 32:19–27 (in Japanese)

Jang J, Chang KS, Park SH, Ji S-J, Lee HJ, Byeon JG, Hwang H-S, Lee YM (2013) Floristic study of Gogunsan Archipelago, Jeollabuk-do, Korea. J Asia Pac Biodivers 6:467–484

Jang J, Park S-H, Chang KS, Ji SJ, Jung SY, Lee HJ, Hwang H-S, Lee Y-M (2013) Diversity of vascular plants in Daebudo and its adjacent regions, Korea. J Asia Pac Biodivers 6:261–280

Jang J-W, Yang J-C, Jung S-Y, Lee H-J, Yun J-E, Chang C, Hwang H-S, Chang K-S, Oh SH, Lee Y-M (2014) The distribution of vascular plants in Banronsan (Mt.) at Jeongseon Gangwon-do, Korea. J Asia Pac Biodivers 7:e30–e39

Jin L, Zhang Y, Yan L, Guo Y, Niu L (2012) Phenolic compounds and antioxidant activity of bulb extracts of six *Lilium* species native to China. Molecules 17:9361–9378

Kang Y, Łuczaj Ł, Ye S, Zhang S, Kang J (2012) Wild food plants and wild edible fungi of Heihe valley (Qinling Mountains, Shaanxi, central China): herbophilia and indifference to fruits and mushrooms. Acta Soc Bot Pol 81:405–413

Kim JH, Kyung HY, Cheoi YS, Lee JK, Hiramatsu M, Okubo H (2006) Geographic distribution and habitat differentiation in diploid and triploid *Lilium lancifolium* of South Korea. J Fac Agric Kyushu Univ 51:239–243

[Kim](https://www.e-kjpt.org/articles/search_result.php?term=author&f_name=Sun%20Yu&l_name=Kim) S-Y, [Moon](https://www.e-kjpt.org/articles/search_result.php?term=author&f_name=Soon%20Hwa&l_name=Moon) S-H, [Kim](https://www.e-kjpt.org/articles/search_result.php?term=author&f_name=Jin%20Seok&l_name=Kim) J-S, [Kim](https://www.e-kjpt.org/articles/search_result.php?term=author&f_name=Jung%20Hyun&l_name=Kim) J-H, [Lee](https://www.e-kjpt.org/articles/search_result.php?term=author&f_name=Byoung%20Yoon&l_name=Lee) BY (2013) First record of *Ajuga nipponensis* Makino (Lamiaceae) from Korea. Korean J Pl Taxon 43:165–167

Kim H-J, Ji S-J, Jung S-Y, Park SH, Lee S-G, Lee C-W, Chang KS (2015) Flora of vascular plants in Deokjeokdo (Ongjin-gun) and its adjacent regions, Korea. Korean J Plant Resour 28:487–510 (in Korean)

Kim JS, Lee IK, Yun BS (2015) A novel biosurfactant produced by *Aureobasidium pullulans* L3-GPY from a tiger lily wild flower, *Lilium lancifolium* Thunb. PLoS ONE 10:e0122917

King WL (1912) The flora of Northampton County, Pennsylvania (Continued). Torreya 12:124–132

Kiselyova AG (2011) Preservation of the biodiversity of vascular plants of sea coasts of Primorskiy territory. In: Structure and dynamics of ecosystems in Siberia and Russian Far East: a collection of scientific papers. Nakhodka: Institute of Technology and Business, pp 144–156 (in Russian)

Knight OW (1906) Some noteworthy plants of the Penobscot valley. Rhodora 8:65–66

Komarov VL (1968) Flora of the USSR, vol IV. Israel Program for Scientific Translations, Jerusalem

Lewington RJ, West CJ (2008) Otari Bioblitz: detailing vascular plants, mosses and liverworts. Wellington Bot Soc Bull 51:5–23

Liang Z, Zhang Y, Li L, Zhang X (2014) Diversity in wild populations of *Lilium lancifolium* native to southern Shaanxi Province. J. Zhejiang A & F Univ 31:885–891

Lunell J (1915) Enumerantur Plantae Dakotae Septentrionalis Vasculares/The Vascular Plants of North Dakota. III. Am Midl Nat 4:229–244

Matsui H (2012) List of vascular plants known from Matsuyama City, Ehime Prefecture, Shikoku, Japan. 2nd version. pp 313–376. In: Committee for Surveys of Natural Environment of Matsuyama City (Chief Editor: Ishikawa, K.). Checklist of the Wild Animals, Fungi, and Plants of Matsuyama City. Department of Environment, Matsuyama City.

Miller NA (1990) A comparison study of two plant communities along the south fork-forked deer river. In: Hamilton SW, Finley MT (eds) Proceedings of the Third Symposium on the Natural History of Lower Tennessee and Cumberland River Valleys. The Center for Field Biology, Austin Peay State University, Clarksville, Tennessee. pp 107–111

Millspaugh CF, White D (1913). West Virginia geological survey. Part I. The living flora of West Virginia. – Part II. The fossil flora of West Virginia Wheeling News Litho. Co., Wheeling.

Nagamitsu T, Nagamasu H (1994) Keys for the pollen of Ashiu, Central Japan. Contr Biol Lab Kyoto Univ 28:261–355

Nichols W, Nichols V (2008) The land use history, flora, and natural communities of the Isles of Shoals, Rye, New Hampshire and Kittery, Maine. Rhodora 110:245–295

Nieuwland J (1913) Notes on our local plants. IV. Am Midl Nat 3:98–125

Noda S (1978) Chromosomes of diploid and triploid forms found in the natural populations of tiger lily in Tsushima. Bot Mag (Tokyo) 91:279–283

Noda S, Hayashi K, Song NH (2003) Detection and spatial dispersion of B chromosome in the triploid form of *Lilium lancifolium*, Liliaceae. Chromosome Sci 7:61–69

Omote J, Yamagiwa Y (2012) Hydrogeochemical investigation of peatlands and related vegetation complexes in Minamidobu and Kitadobu mires in central Japan. Finn Environ 28:349–366

Overbeck C (2012) A walk at Clifton Farms. Leaflet Autumn 2012:1

Poindexter DB (2013) Vascular flora and plant communities of Alleghany County, North Carolina. J Bot Res Inst Texas 7:529–574

Rhoads AF, Klein WM (1993) The vascular flora of Pennsylvania: annotated checklist and atlas, vol 207. American Philosophical Society, Philadelphia

Robinson J (1880) The Flora of Essex County, Massachusetts. Essex Institute, Salem.

Sato M, Shida Y (2013) A flora on tumuli in Kutsugata District, Rishiri Island, Hokkaido (2). Rishiri Stud 32:63–69 (in Japanese)

Shao X-B, Zhou X, Xu Y, Zhao T-L, Zhu P-B, Liu X-M (2010) Study on tissue culture of *Lilium lancifolium* Thumb, wild lily. Tianjin Agric Sci 16(4): 18–19

Shin HT, Hwang JH, Yoon KJ (2011) Distribution of vascular plants in the Ulleung forest trail area (Seokpo to Naesujeon). J Korean Nat 4:79–85

Shirakawa K, Okonogi H, Fukuda K (2014) Habitat characteristics and conservation of rare herbaceous species in a suburban forest around Konbukuro pond in Kashiwa city, Chiba prefecture. Rep Chiba Biodivers Cent 7:14–27 (in Japanese)

Singhurst JR, Holmes WC (2010) *Lilium lancifolium* (Liliaceae): new to Texas. Phytologia 92:56–58

Smith PF, Woodland DW (2006) Vascular plant study of Warren Dunes State Park, Berrien County, Michigan. Mich Bot 45:1–58.

Smyth BB (1889) Additions to the flora of Kansas. Trans Kans Acad Sci 12 (1889–1890): 105–119

Son H-D, Gwon S-G, Jang J-W, Sun E-M, Kim B-A, Im H-T (2013) Floristic study of Jang-do (Isl.) in Korea*.* J Spec Res 2: 227–244.

Suiter DW, Evans DK (1999) Vascular flora and rare species of New River Gorge National River, West Virginia. Castanea 64:23–49

Susaj E, Susaj L, Pazari F (2011) Lily (*Lilium* spp.) – an important plant of wild and cultivated flora in Tirana district. VII-th International Symposium: “Biodiversity: Conservation and Sustainable Use for Rural Development”, Tirana, 30 September 2011, At Tirana, Albania, vol 1, pp. 155–160 (in Albanian)

Takahashi A (2011) A List of vascular plants in Sanda City, Hyogo Prefecture. Hum Nat 22:101–146 (in Japanese)

Truong NX, Lee S-I, Rai R, Kim N-S, Kim JH (2016) Ribosomal DNA locus variation and REMAP analysis of the diploid and triploid complexes of *Lilium lancifolium*. Genome 59:551–564

Tsutaya T, Sawada J, Dodo Y, Mukai H, Yoneda M (2013) Isotopic evidence of dietary variability in subadults at the Usu-moshiri site of the Epi-Jomon culture, Japan. J Archaeol Sci 40:3914–3925

Vrishch DL (2011) History and prospects for study of lily wild species biodiversity in the Russian Far East. Vestnik KrasGAU 2011(1):53–57

Zhi L, Teng Z, Li X, Sui S, Li M (2011) Phylogenetic relationship analysis of 23 wild species of *Lilium* by SRAP markers. J Agric Biotechnol 19:677–684 (in Chinese)

**Online documents with *Lilium lancifolium* occurrences**

AECOM (2013) Sunnidale Road - Highway 400. Watermain Crossing Municipal Class. Environmental Assessment. Project File Report. <http://www.barrie.ca/Living/Environment/Documents/Sunnidale%20Road-Hwy%20400%20WM%20Crossing%20-%20Appendix%20F.pdf>

Angelo R (2014) Vascular Flora of Concord, Massachusetts. <http://www.ray-a.com/ConcordMassFlora.pdf>

BioLogic (2012) Natural Heritage Study. <http://www.elgincounty.ca/sites/default/files/u235/PDF%20Documents/BioLogic_Elgin-CR24_NHS_final-report2.pdf>

Cooney PL (2001) Clarence Fahnestock (Canopus Lake). <http://nynjctbotany.org/lgtofc/nycanops.html>

Cromartie J (2012) Changing Flora of the Stockton College Campus 1970-2012. Pinelands Research Series. https://www.nj.gov/pinelands/science/pinesseries/Jamie%20Cromartie%20-%20Changing%20Flora%20of%20the%20Stockton%20College%20Campus%201970-2012.pdf

Dawood Engineering. Inc. (2006) Wetland identification and delineation report. <http://www.dotdom1.state.pa.us/jpa%5CjpaApplications.nsf/ByUNID/FB68CA35E0773B828525744E00569F5B/$File/SR1008_WetlandReport.pdf>

Edwards MT, Edwards EP (1991) An annotated list of the wild vascular plants of the Sweet Briar College property. Sweet Briar College Natural History Series 2. https://www.yumpu.com/it/document/view/11518244/sweet-briar-campus-plant-list-natural-history-at-sbc-sweet-briar-/7

Goodwin CE, A List of the Vascular Plants of Northumberland County, Ontario. <http://www.willowbeachfieldnaturalists.org/assets/bird-assets/downloads/Plants.pdf>

Hazlet BT (1986) The Terrestrial Vegetation and Flora of the Mainland Portion of Sleeping Bear Dunes National Lakeshore. <https://deepblue.lib.umich.edu/bitstream/handle/2027.42/49255/1838379.0013.001.pdf>

Horton D (2011) Flora of Rochester Cemetery. <http://bio.cgrer.uiowa.edu/herbarium/CheckLists/Rochester%20Cemetery%20Plant%20List%20by%20Species%20Common%20May%202011.pdf>

Issuance Saitama-ken (2010) Report on conservation management around fountain of Yatta. <http://www.city.iruma.saitama.jp/_res/projects/default_project/_page_/001/002/022/yata_houkokusyo.pdf>

Local & Traditional Knowledge for Wise Use of Resources in Satoyama Villages In Nashino, Onosechou, Toyota City <http://chubu.env.go.jp/to_2010/data/1019a_11.pdf>

MDC Survey (2002) Peddocks Island plants. <https://www.nps.gov/boha/learn/nature/upload/PEDDOCKS-ISLAND-PLANTS.pdf>

Noto Peninsula Satoyama-Satoumi Nature School (2009) Preliminary Survey of Biodiversity in Noto Peninsula Satoyama-Satoumi Landscapes. <http://www.satoyama-satoumi.com/houkoku2008/shiryo/kenkou.pdf>

OEL-HydroSys (2012) Bonnechere River proposed Thomas Low Waterpower project. <http://www.renfrewpg.ca/wp-content/uploads/2015/08/AnnexII.pdf>

Oldham MJ (2010) Checklist of the Vascular Plants of Niagara Regional Municipality, Ontario. <https://www.researchgate.net/publication/274252640_Checklist_of_the_Vascular_Plants_of_Niagara_Regional_Municipality_Ontario>

US Fish and Wildlife Service (2005) Assabet River National Wildlife Refuge. Final Comprehensive Conservation Plan. <https://www.fws.gov/uploadedFiles/Region_5/NWRS/North_Zone/Eastern_Massachusetts_Complex/Assabet_River/AssabetFinalCCP.pdf>

US Fish and Wildlife Service (2005) Detroit River International Wildlife Refuge / Comprehensive Conservation Plan. <https://www.fws.gov/midwest/planning/detroitriver/finalCCP/DetroitRiver_finalCCP.pdf>

Waikato Botanical Society Inc. Karangahake Gorge fieldtrip 2nd October 2005. Newsletter 21. <http://waikatobotsoc.org.nz/newsletters/WaiBotSoc%20Newsletter%2021.%20Nov%202005.pdf>

White DJ (2016) Plants of Lanark County Ontario. <http://www.lanarkflora.com/Lanark_plants_revised_2016.pdf>

**Appendix B: Tables and Figures**

**Table S1. Chromosome counts of *Lilium lancifolium* found in the literature with the indication of the samples origin.**

| Area | Country | Province^1^ | Chromosome count (2*n*) | Reference |
| --- | --- | --- | --- | --- |
| ASIA | China | Heilongjiang | 36 | Noda (1986); Yang et al. (1997)^2^; Rong et al. (2011) |
| ASIA | China | Jiangsu | 36 | Zhong et al. (1993)^2^; Truong et al. (2016) |
| ASIA | China | Jilin | 36 | Tolgor and Liu (1996); Rong et al. (2011) |
| ASIA | China | Liaoning | 36 | Rong et al. (2011); Truong et al. (2016) |
| ASIA | China | Shangdong | 36 | Truong et al. (2016) |
| ASIA | China | Sichuan | 36 | Gao et al. (2009, 2011) |
| ASIA | China | Zhejiang | 36 | Truong et al. (2016) |
| ASIA | Japan | –^3^ | 36 | Noda (1986) |
| ASIA | Japan | Fukui | 36 | Truong et al. (2016) |
| ASIA | Japan | Fukuoka | 36 | Truong et al. (2016) |
| ASIA | Japan | Hiroshima | 36 (37) | Noda et al. (2003) |
| ASIA | Japan | Nara | 36 (37) | Noda et al. (2003) |
| ASIA | Japan | Tottori | 36 | Truong et al. (2016) |
| ASIA | Japan | Toyama | 36 | Truong et al. (2016) |
| ASIA | Japan | Yamaguchi | 36 | Truong et al. (2016) |
| ASIA | Russia | Primorsky | 24, 36 | Probatova et al. (2001)*; Sun et al. (2002)** |
| EUR | Romania | – | 36 | Noda (1986)^2^ |
| Native | Japan | Tsushima | 24, 36 | Noda (1978, 1986); Noda and Hayashi (1989)*; Truong et al. (2016) |
| Native | Korea | – | 24, 36 | Noda (1986) |
| Native | Korea | Busan | 24 | Kim et al. (2006b) |
| Native | Korea | Chungcheongbuk-do | 36 | Song et al. (2012); Truong et al. (2016) |
| Native | Korea | Chungcheongnam-do | 24, 36 | Kim et al. (2006a, 2006b); Shim et al. (2007); Song et al. (2012)**; Truong et al. (2016)** |
| Native | Korea | Dok-do^4^ | 36 | Sun et al. (2002) |
| Native | Korea | Gangwon-do | 36 | Kim et al. (2006a, 2006b); Shim et al. (2007); Song et al. (2012); Truong et al. (2016) |
| Native | Korea | Gyeonggi-do | 36 | Kim et al. (2006a, 2006b); Shim et al. (2007); Song et al. (2012) |
| Native | Korea | Gyeongsangbuk-do | 36 | Kim et al. (2006a, 2006b); Shim et al. (2007); Song et al. (2012); Truong et al. (2016) |
| Native | Korea | Gyeongsangnam-do | 24, 36 | Kim et al. (2006b)*; Shim et al. (2007)*; Song et al. (2012)**; Truong et al. (2015*, 2016*) |
| Native | Korea | Incheon | 24, 36 | Kim et al. (2006a, 2006b); Shim et al. (2007); Song et al. (2012); Truong et al. (2015*, 2016) |
| Native | Korea | Jeju-do | 24, 36 | Sun et al. (2002)**; Kim et al. (2006a**, 2006b); Shim et al. (2007)**; Song et al. (2012)**; Truong et al. (2016) |
| Native | Korea | Jeollabuk-do | 24, 36 | Kim et al. (2006a*, 2006b*); Shim et al. (2007)*; Song et al. (2012)**; Truong et al. (2016)** |
| Native | Korea | Jeollanam-do | 24, 36 | Sun et al. (2002)*; Kim et al. (2006a, 2006b); Shim et al. (2007); Song et al. (2012)**; Truong et al. (2015*, 2016); Chung et al. (2016)** |
| Native | Korea | Ulleung-do^4^ | 36 | Sun et al. (2002); Kim et al. (2006b); Shim et al. (2007); Truong et al. (2016) |
| Native | Korea | Ulsan | 24 | Kim et al. (2006b); Truong et al. (2016) |
| USA-CA | USA | Ohio | 36 | Westfall (1940)^2^ |
| USA-CA | USA | Maryland | 36 | Stewart and Bamford (1943) |
| USA-CA | USA | New York?^5^ | 36 | Chandler et al. (1937) |
| USA-CA | USA | Pennsylvania | 36 | Stewart and Bamford (1943) |
| USA-CA | USA | – | 36 | Stewart and Bamford (1943)^2^; Noda (1986)^2^ |

^1^ Or other equivalent first-level administrative division.

^2^ Cultivated population/s.

^3^ Studied populations came from the four main Japanese islands (Hokkaido, Honshu, Kyushu, and Shikoku), but with no indication of the prefecture.

^4^ Despite belonging to Gyeongsangbuk-do Province, the island is shown separately due to its long distance from the Korean Peninsula.

^5^ There is no information on the source of the material, but it was probably cultivated at the New York Botanical Garden, as two of the authors were established there at the time of the article’s publication. According to the authors, the triploid plants observed were “from the type clone of the triploid *Lilium tigrinum* which was first sent from China to the Royal Botanic Gardens at Kew, England, by William Kerr in 1804 and propagated since solely by asexual means for horticultural culture” (Chandler *et al*. 1937: 757).

*Only cited the diploid cytotype.

**Only cited the triploid cytotype.

**References**

Chandler C, Porterfield WM, Stout AB (1937) Microsporogenesis in diploid and triploid types of *Lilium tigrinum* with special references to abortions. Cytologia Fujii jub vol: 756-784

Chung G-Y, Nam B-M, Choi M-J, Jang H-D, Choi H-J, Oh B-U (2016) Chromosome numbers of 50 vascular plants in South Korea. J Asia-Pac Biodivers 9:496–504

Gao Y-D, Zhou S-D, He X-J (2009) Karyotypes of four genera in Liliaceae (s. str.) from Hengduan Mountains of southwestern China. Acta Bot Yunnan 31:399–405 (in Chinese)

Gao Y-D, Zhou S-D, He X-J (2011) Karyotype studies in thirty-two species of *Lilium* (Liliaceae) from China. Nord J Bot 29:746–761

Kim JH, Jang WS, Kyung HY, Xuan Y, Davaasuren YE, Sim EJ, Lee JK, Choi YS, Hiramatsu M, Kim KW, Yoo K-O (2006a) A principal component analysis for the morphological characters of diploid and triploid populations of *Lilium lancifolium* in Korea. Korean J Plant Resour 19:300–307 (In Korean)

Kim JH, Kyung HY, Choi YS, Lee JK, Hiramatsu M, Okubo H (2006b) Geographic distribution and habitat differentiation in diploid and triploid *Lilium lancifolium* of South Korea. J Fac Agric Kyushu Univ.51:239–243

Noda S (1978) Chromosomes of diploid and triploid forms found in the natural populations of tiger lily in Tsushima. Bot Mag (Tokyo) 91:279–283

Noda S (1986) Cytogenetic behavior, chromosomal differentiations, and geographic distribution in *Lilium lancifolium* (Liliaceae). Plant Spec Biol 1:69–78

Noda S, Hayashi K (1989) Cytogenetic study and a record on the natural occurrences and habitats of *Lilium lancifolium* var. *flaviflorum* in Tsushima, Japan. Bull Cult Nat Sci Osaka Gakuin Univ 20:21–43 (in Japanese)

Noda S, Hayashi K, Song NH (2003) Detection and spatial dispersion of B chromosome in the triploid form of *Lilium lancifolium*, Liliaceae. Chromosome Sci 7:61–69

Probatova NS, Rudyka EG, Shatalova SA (2001) Chromosome numbers in some plant species from the environs of Vladivostok city (Primorsky Region). Bot. Zhurn 86(1):168–172 (in Russian)

Rong L, Lei J, Wang C (2011) Collection and evaluation of the genus *Lilium* resources in Northeast China. Genet Resour Crop Evol 58:115–123

Shim EJ, Lee JK, Choi YS, Kim JH (2007) Analysis of genetic variation in diploid and triploid populations of *Lilium lancifolium* Thumb. native to Korea using RAPD markers. Flower Res J 15:224–231 (in Korean)

Song YS, Truong NX, Kim NS, Kim JH (2012) Analysis of genetic variation in native Korean tiger lily (*Lilium lancifolium* Thunb.) by EST–SSRs. Flower Res J 20:75–82 (in Korean)

Stewart RN, Bamford R (1943) The nature of polyploidy in *Lilium tigrinum*. Am J Bot 30:1–7

Sun B-Y, Sul MR, Im JA, Kim CH, Kim TJ (2002) Evolution of endemic vascular plants of Ulleungdo and Dokdo in Korea – floristic and cytotaxonomic characteristics of vascular flora of Dokdo. Korean J Pl Taxon 32:143–158 (in Korean)

Tolgor B, Liu LB (1996) Studies of karyotypes of 5 species in *Lilium* from Jilin. J Wuhan Bot Res 14:6–12 (in Chinese)

Truong NX, Song Y-S, Kim N-S, Park J-W, Kim J-H, Wakana A (2015) Occurrence and survival of autotriploids in natural diploid populations of *Lilium lancifolium* Thunb. J Fac Agr Kyushu Univ 60:73–80

Truong NX, Lee S-I, Rai R, Kim N-S, Kim JH (2016) Ribosomal DNA locus variation and REMAP analysis of the diploid and triploid complexes of *Lilium lancifolium*. Genome 59:551–564

Westfall JJ (1940) Cytological studies of *Lilium tigrinum*. Bot Gaz 101:550–581.

Yang L-P, Liu X-M, Zhang X-F (1997) Study on cytology of *Lilium lancifolium*. Bull Bot Res 17:85–88 (In Chinese)

Zhong YQ, Li HB, Wang KJ (1993) Analysis of karyotype and C-banding pattern of Zijinshan lily. Jiangsu J Agr Sci 9:8–12 (in Chinese)

**Table S2. Descriptive statistics of the selected variables for each area, and results from the overall test for difference between groups (*p*-value; *p*) and of pair-wise comparisons of means.** Only significant differences found in the pairwise comparisons are reported. *X* = mean value; SD = standard deviation; Min = minimum value; Max = maximum value. Values in brackets are outliers of the common interval of values. Number of occurrences (*N*): Native (*N* = 104), ASIA (*N* = 318), AUS-NZ (*N* = 32), EUR (*N* = 72), USA-CA (*N* = 201).

| **Variable** | **Area** | ***X*** | **SD** | **Min** | **Max** | ***p*-value** | **Significant differences in**  ***post-hoc* test (Games-Howell)** |
| --- | --- | --- | --- | --- | --- | --- | --- |
| Bio2 (Mean  Diurnal  Range; ºC) | Native | 8.99 | 1.62 | (6)8.7 | 9.3(11.9) | 0.000 | EUR < Native/ASIA/AUS-NZ/USA-CA *p* = 0.000  USA-CA > Native/ASIA/AUS-NZ *p* = 0.000  Native > ASIA *p* = 0.011 |
|  | ASIA | 8.51 | 1.35 | (5.8)8.4 | 8.6(15.5) |  |  |
|  | AUS-NZ | 8.88 | 0.94 | (6.6)8.5 | 9.2(11.6) |  |  |
|  | EUR | 7.10 | 1.12 | (4.6)6.8 | 7.4(12.8) |  |  |
|  | USA-CA | 11.44 | 1.11 | (7.6)11.3 | 11.6(14.1) |  |  |
| Bio5 (Max Temperature  of Warmest Month; ºC) | Native | 28.71 | 1.38 | (23.7)28.4 | 29(30.4) | 0.000 | Native > ASIA *p* = 0.032  Native > AUS-NZ/EUR *p* = 0.000  ASIA > AUS-NZ/EUR *p* = 0.000  USA-CA > AUS-NZ/EUR *p* = 0.000 |
|  | ASIA | 27.83 | 3.33 | (19.3)27.4 | 28.2(34.2) |  |  |
|  | AUS-NZ | 21.14 | 1.97 | (18.2)20.4 | 21.8(25.2) |  |  |
|  | EUR | 20.76 | 1.29 | (15.1)20.5 | 21.1(23.9) |  |  |
|  | USA-CA | 28.03 | 2.22 | (23)27.7 | 28.3(35.1) |  |  |
| Bio11 (Mean Temperature  of Coldest Quarter; ºC) | Native | 0.47 | 3.30 | (-6.9)-0.2 | 1.1(7.3) | 0.000 | Native > EUR/USA-CA *p* = 0.000  AUS-NZ > Native/ASIA/EUR/USA-CA *p* = 0.000  ASIA > EUR/USA-CA *p* = 0.000 |
|  | ASIA | 0.93 | 5.09 | (-14.8)0.4 | 1.1(12.7) |  |  |
|  | AUS-NZ | 6.71 | 2.23 | (0.4)5.9 | 7.5(11.2) |  |  |
|  | EUR | -3.28 | 3.08 | (-13.2)-4.0 | -2.6(1.9) |  |  |
|  | USA-CA | -3.35 | 4.50 | (-16.0)3.9 | -2.7(7.9) |  |  |
| Bio12 (Annual Precipitation; mm) | Native | 1326.19 | 257.55 | (703)1276 | 1376(2162) | 0.000 | AUS-NZ > Native/ASIA/EUR/USA-CA *p* = 0.000  EUR < Native/ASIA/USA-CA/AUS-NZ *p* = 0.000  USA-CA < Native/ASIA/AUS-NZ *p* = 0.000 |
|  | ASIA | 1308.37 | 414.13 | (421)1263 | 1354(2962) |  |  |
|  | AUS-NZ | 1901.00 | 1029.24 | (500)1530 | 2272(4262) |  |  |
|  | EUR | 656.46 | 181.14 | (454)614 | 699(1657) |  |  |
|  | USA-CA | 1053.49 | 184.68 | (438)1028 | 1079(1538) |  |  |
| Bio 15  (Precipitation Seasonality; coefficient of variation) | Native | 72.13 | 16.33 | (25)69 | 75(103) | 0.000 | Native > ASIA/AUS-NZ /EUR/USA-CA *p* = 0.000  ASIA > AUS-NZ /EUR/USA-CA *p* = 0.000  EUR > USA *p* = 0.001  EUR > AUS-NZ *p* = 0.025 |
|  | ASIA | 52.20 | 22.51 | (20)50 | 55(148) |  |  |
|  | AUS-NZ | 16.78 | 5.17 | (9)15 | 19(32) |  |  |
|  | EUR | 28.08 | 8.37 | (21)26 | 30(91) |  |  |
|  | USA-CA | 18.60 | 12.05 | (6)17 | 20(59) |  |  |

**Table S3. Descriptive statistics of the principle components analysis (PCA) of the selected variables.** In bold the variables that mainly explained each axis.

| Environmental variable | PC1 | PC2 | PC3 |
| --- | --- | --- | --- |
| bio2 | -0.197 | 0.832 | -0.418 |
| bio5 | 0.635 | 0.674 | -0.047 |
| bio11 | 0.885 | -0.166 | -0.023 |
| bio12 | 0.748 | -0.262 | -0.318 |
| bio15 | 0.237 | 0.335 | 0.872 |
| Eigenvalue | 1.841 | 1.355 | 1.039 |
| Explained variance (%) | 36.82 | 27.11 | 20.78 |

**Table S4. Test of niche divergence vs. conservatism on each principal component (PC) between pair comparisons.** Note that for niche conservatism (C), *d*_n_ should be smaller than *d*_b_, whereas for niche divergence (D) *d*_n_ should be significant itself and greater than *d*_b_ (McCormack et al., 2010). Not conclusive of niche divergence or niche conservatism (NC) is applied when *d*_n_ = *d*_b_ or *d*_n_ is greater than *d*_b_, but *d*_n_ is not significant itself. Explained percentage of variance, most contributed variables and a biological explanation are also provided.

|  |  | ***d*_n_** | ***d*_b_ (95%CIs)** | **Niche pattern** | **% variance explained** | **Most important variables** | **Biological interpretation** |
| --- | --- | --- | --- | --- | --- | --- | --- |
| **Native**  **vs.**  **ASIA** | PC1 | 0.214 ns | 0.427  (0.324, 0.526) | **C** | 45.66% | bio11, bio12 | min. temp. / rainfall |
|  | PC2 | 0.926 ns | 0.612  (0.511, 0.709) | NC | 32.41% | bio5, bio15 | max. temp. / rainfall |
|  | PC3 | 0.680* | 0.612  (0.514, 0.709) | NC | 10.83% | bio2, bio12 | range temp. / rainfall |
| **Native**  **vs.**  **USA-CA** | PC1 | 1.685* | 1.411  (1.340, 1.492) | **D** | 46.04% | bio11, bio12 | min. temp. / rainfall |
|  | PC2 | 0.852* | 1.144  (1.057, 1.231) | **C** | 26.24% | bio2, bio5 | range & max. temp. |
|  | PC3 | 0.817* | 0.727  (0.623, 0.825) | NC | 16.04% | bio12, bio15 | rainfall |
| **Native**  **vs.**  **AUS-NZ** | PC1 | 1.670* | 1.735  (1.685, 1.788) | **C** | 57.50% | bio5, bio11 | max. & min temp. |
|  | PC2 | 1.146* | 0.139  (0.028, 0.241) | **D** | 21.74% | bio2, bio5 | range & max. temp. |
|  | PC3 | 0.827 ns | 0.536  (0.353, 0.727) | NC | 13.51% | bio12 | rainfall |
| **Native**  **vs.**  **EUR** | PC1 | 1.971* | 1.965  (1.942, 1.990) | NC | 65.50% | bio5, bio12, bio15 | max. temp. / rainfall |
|  | PC2 | 0.018 ns | 0.004  (-0.110, 0.107) | NC | 27.64% | bio2, bio11 | range & min. temp. |
|  | PC3 | 0.990 ns | 0.051  (-0.159, 0.061) | NC | 5.66% | bio12 | rainfall |
| **ASIA**  **vs.**  **USA-CA** | PC1 | 1.160* | 1.011  (0.936, 1.099) | **D** | 43.19% | bio11, bio12 | min. temp. / rainfall |
|  | PC2 | 1.284* | 1.492  (1.429, 1.556) | **C** | 25.19% | bio2, bio5 | range & max. temp. |
|  | PC3 | 1.084* | 0.369  (0.275, 0.467) | **D** | 21.98% | bio15 | rainfall |
| **ASIA**  **vs.**  **AUS-NZ** | PC1 | 1.328* | 1.472  (1.407, 1.541) | **C** | 41.12% | bio12, bio15 | rainfall |
|  | PC2 | 0.988* | 0.759  (0.671, 0.845) | **D** | 30.04% | bio5, bio11 | max. & min  temp. |
|  | PC3 | 2.014* | 0.905  (0.823, 0.990) | **D** | 14.14% | bio2 | range temp. |
| **ASIA**  **vs.**  **EUR** | PC1 | 1.995* | 1.737  (1.691, 1.782) | **D** | 46.47% | bio5, bio11 | max. & min  temp. |
|  | PC2 | 0.840* | 0.231  (0.139, 0.322) | **D** | 34.58% | bio2, bio15 | range temp. / rainfall |
|  | PC3 | 0.172 ns | 0.452  (0.359, 0.546) | **C** | 9.63% | bio12 | rainfall |

* Denotes significance (*p* < 0.05).

ns Denotes no significance (*p* ≥ 0.05).

**Figure S1. Curve responses of presences (in colour) and background (in gray) points in each area of 26 layers that may potentially influence *Lilium lancifolium* distribution.** Density of points in axis Y and variables in axis X. Variables: Altitude, Aspect, Bio1 (Annual Mean Temperature), Bio2 (Mean Diurnal Range), Bio3 (Isothermality), Bio4 (Temperature Seasonality), Bio5 (Max Temperature of Warmest Month), Bio6 (Min Temperature of Coldest Month), Bio7 (Temperature Annual Range), Bio8 (Mean Temperature of Wettest Quarter), Bio9 (Mean Temperature of Driest Quarter), Bio10 (Mean Temperature of Warmest Quarter), Bio11 (Mean Temperature of Coldest Quarter), Bio12 (Annual Precipitation), Bio13 (Precipitation of Wettest Month), Bio14 (Precipitation of Driest Month), Bio15 (Precipitation Seasonality), Bio16 (Precipitation of Wettest Quarter), Bio17 (Precipitation of Driest Quarter), Bio18 (Precipitation of Warmest Quarter), Bio19 (Precipitation of Coldest Quarter), compound topographic index (CTI), Flow accumulation, Flow direction, Human Foot Print (HFP), and Slope.

**
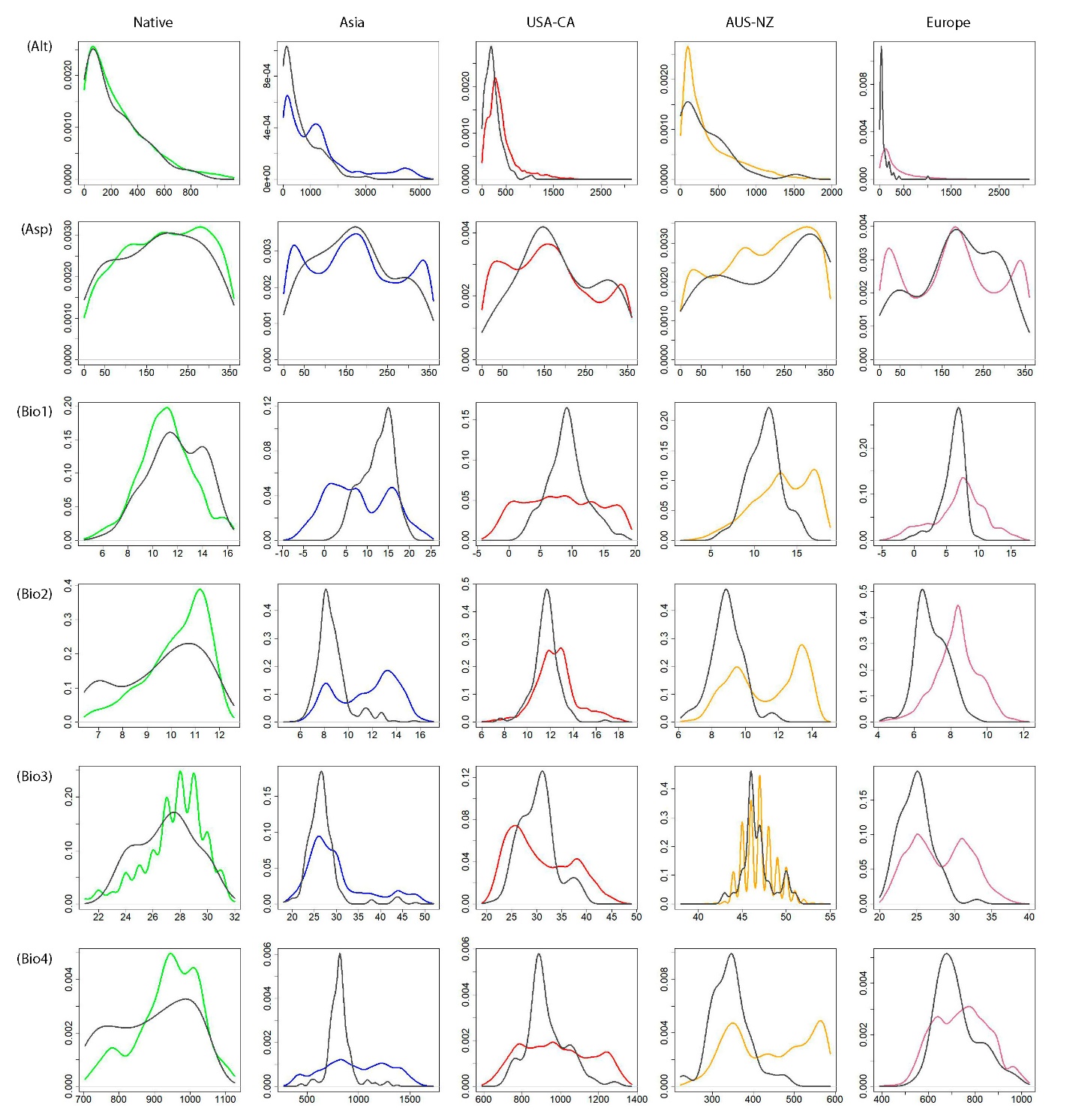
**

**
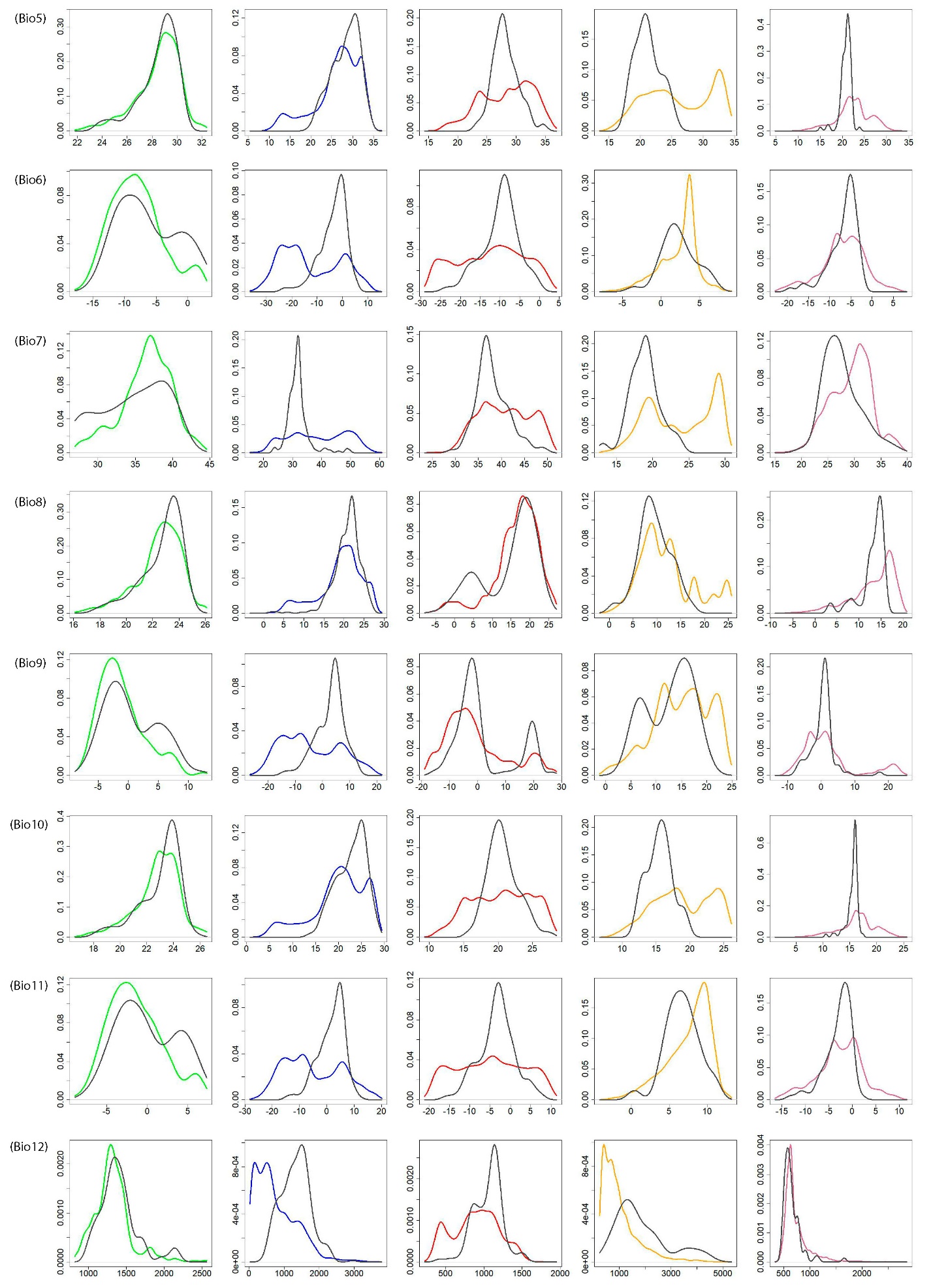
**

**
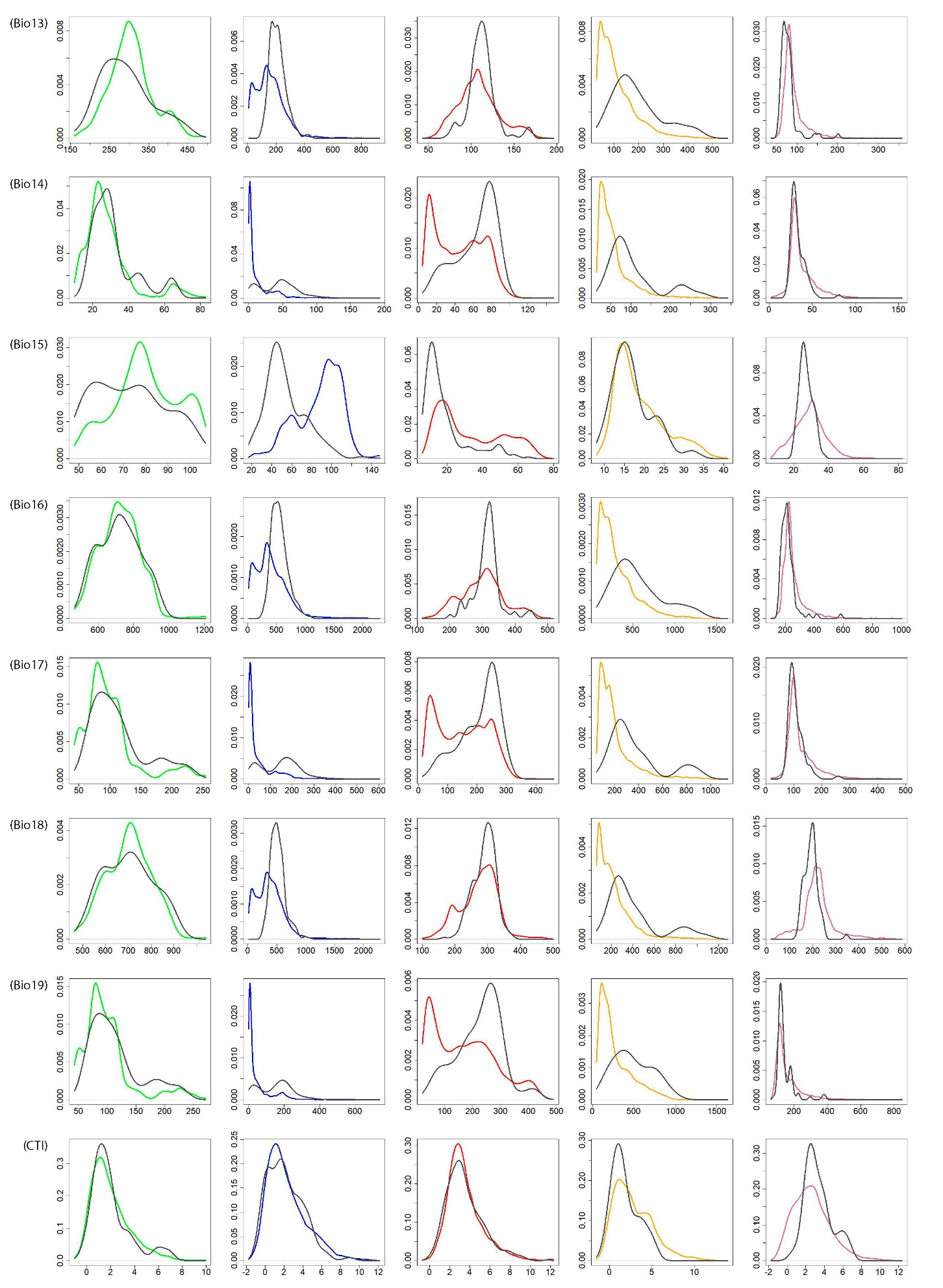
**

**
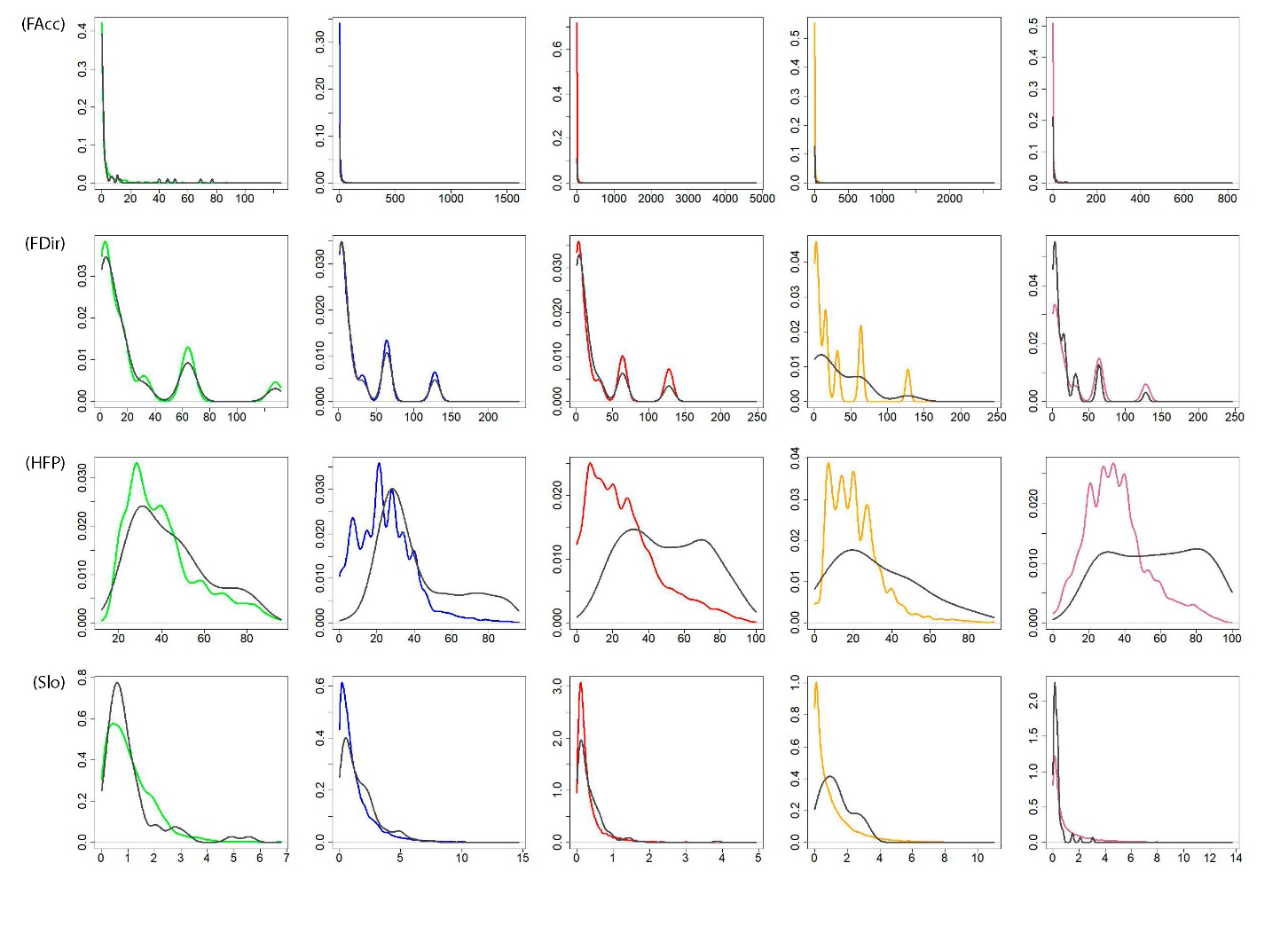
**

**Figure S2. Contribution of each environmental variable to spatial distribution of the PCA-env.**

**
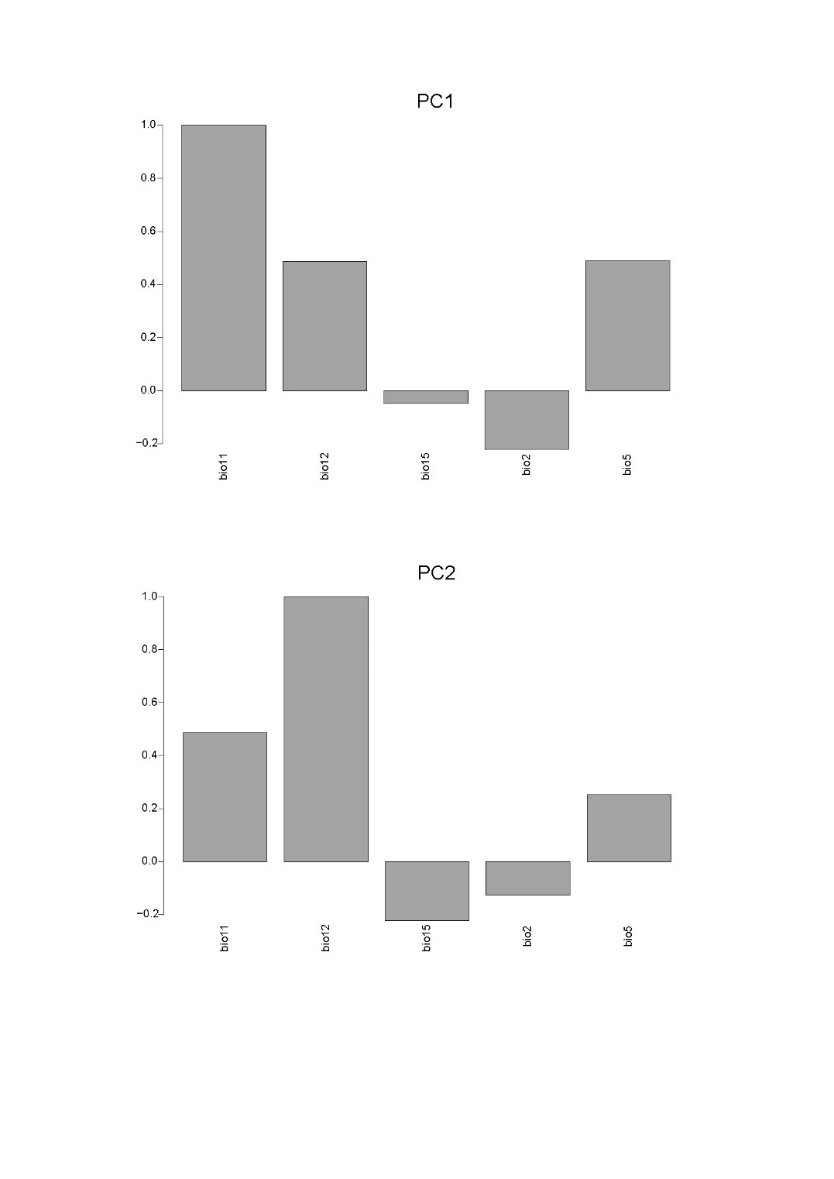
**

**Figure S3. Video of the minimum-volume ellipsoids (MVE) of different regions into the 3D environmental space performed with NicheA.**

**Appendix C: R script applied (modified from Silva et al. 2016)**

**Silva DP, Vilela B, Buzatto BA, Moczek AP, Hortal J** (2016) Contextualized niche shifts upon independent invasions by the dung beetle *Onthophagus taurus*. Biol Invasions 18:3137–3148

# ---------------- Code used in "Climatic niche evolution among native and introduced areas (as an archaeophyte and neophyte) of Lilium lancifolium (Liliaceae)" ----------------

# ---------------- Installation of packages ----------------

install.packages("BIOMOD",repos="http://R-Forge.R-project.org")

install.packages("ade4")

install.packages("adehabitat")

install.packages("sp")

install.packages("gam")

install.packages("MASS")

install.packages("mvtnorm")

install.packages("gbm")

install.packages("dismo")

install.packages("knitr")

install.packages("spThin")

install.packages("rgeos")

install.packages("maptools")

install.packages("raster")

install.packages("ecospat")

install.packages("spThin")

install.packages("rgdal")

install.packages("riverplot")

# ---------------- Importation of libraries ----------------

library(BIOMOD)

library(ade4)

library(adehabitat)

library(sp)

library(gam)

library(MASS)

library(mvtnorm)

library(gbm)

library(dismo)

library(knitr)

library(spThin)

library(rgeos)

library(maptools)

library(raster)

library(ecospat)

library(spThin)

library(rgdal)

library(riverplot)

# ---------------- Parameters to modify by the user ----------------

# Set working directory

main.directory <- "E:/Lilium lancifolium/TEXT/Supplementary/RCode"

#Defining groups (regions) to be tested and the colors for each region

n.groups <- 5

g.names <- c("Native","Asia","AUS-NZ","Europe","USA-CA")

g.codenames <- c("Native","Asia","AUS-NZ","Europe","USA-CA")

g.colors <- c("green", "blue", "orange","pink3", "red" )

# Import occurrences into data frames

setwd(main.directory)

occ.points.1 <- read.table("occurrences_native.txt", sep = "\t", header = TRUE)

occ.points.2 <- read.table("occurrences_asia.txt", sep = "\t", header = TRUE)

occ.points.3 <- read.table("occurrences_australia.txt", sep = "\t", header = TRUE)

occ.points.4 <- read.table("occurrences_europe.txt", sep = "\t", header = TRUE)

occ.points.5 <- read.table("occurrences_usa.txt", sep = "\t", header = TRUE)

# Join the occurrences into a list of data frames

occ.points <- list(occ.points.1,occ.points.2,occ.points.3,occ.points.4,occ.points.5)

# Import climatic variables information

variables.file.type <- '.bil'

variables.path <- "./variables"

# Offset that the minimum convex polygon (MCP) will apply to create the polygon

buffer.size <- 0.3

#Environmental space: An environmental space is generated based on the PCA values calculated for the background and the occurrence records. We defined the resolution of this two-dimensional space grid below

R <- 500

#Niche overlap: For the niche overlap, we calculate the D metric and its significance, using a similarity test. We define the number of interactions for the similarity test below (see the methods section in the manuscript for details)

rep <- 100

# ---------------- Preparation and visualization of the occurrence points ----------------

# Thinning the occurrences. To reduce sampling bias, the occurrences are trimmed to ensure that a minimum distance of 5 km is lead between one to each other, so modify the parameter "thin.par" according to your study

occ.points.thin <- list()

for (i in 1:n.groups){

occ.points.thin[i] <- thin(occ.points[[i]], verbose = FALSE,

lat.col = "latitude",

long.col = "longitude",

spec.col = "species",

thin.par = 5,

reps = 1,

write.files = FALSE,

write.log.file = FALSE,

locs.thinned.list.return = TRUE,

out.dir = getwd(),

out.base = "ThinProcess")

}

#To check the distribution of the occurrence records we map them in a world context

data(wrld_simpl)

plot(wrld_simpl)

for (i in 1:n.groups){

points(occ.points.thin[[i]]$Longitude, occ.points.thin[[i]]$Latitude , col = g.colors[i], pch = 20, cex = 0.7)

}

#Next, we checked the number of occurrence records per region

number.occ <- list()

for (i in 1:n.groups) {

number.occ[[i]] <- c(g.names[i],nrow(occ.points.thin[[i]]))

}

print(number.occ)

# ---------------- Importation and Visualization of variables ----------------

# Generate the paths vector to read

variables.file.pattern <- paste('*',variables.file.type, sep="")

list.variables<- list.files(path = variables.path, pattern = variables.file.pattern, full.names=TRUE)

# Import each variable file and store them in variables

variables <- stack(list.variables)

# Plot of the variables

plot(variables)

# ---------------- Creation of polygons and their associated data (occurrences and background per region) ----------------

# Define of the Minimum Convex Polygon (MCP) for each region based on their occurrences. This part of the code will take several minutes

# Function wich creates the MCP

mcp <- function (xy) {

xy <- as.data.frame(coordinates(xy))

coords.t <- chull(xy[, 1], xy[, 2])

xy.bord <- xy[coords.t, ]

xy.bord <- rbind(xy.bord[nrow(xy.bord), ], xy.bord)

return(SpatialPolygons(list(Polygons(list(Polygon(as.matrix(xy.bord))), 1))))

}

# Union of the world map

lps <- getSpPPolygonsLabptSlots(wrld_simpl)

IDFourBins <- cut(lps[,1], range(lps[,1]), include.lowest=TRUE)

world <- unionSpatialPolygons(wrld_simpl, IDFourBins)

# Empty objects

xy.mcp <- list() # List of Polygon data for each region

back.env <- list() # List of Background data for each region

spec.env <- list() # List of Occurrences data for each region

# Plot map

plot(wrld_simpl)

# Loop to create the MCP for each region

for(i in 1:n.groups) {

# Background polygon creation

mcp.occ <- mcp(occ.points.thin[[i]]) # Creation of the MCP without buffer

xy.mcp.i <- gBuffer(mcp.occ, width = buffer.size) # Application of the buffer size

proj4string(xy.mcp.i) <- proj4string(world)

xy.mcp[[i]] <- gIntersection(xy.mcp.i, world, byid=TRUE, drop_lower_td=TRUE)

# Background environment

back.env[[i]] <- na.exclude(do.call(rbind.data.frame, extract(variables, xy.mcp[[i]])))

# Species environment

spec.env[[i]] <- na.exclude(extract(variables, occ.points.thin[[i]]))

# Plot of the occurrences and the resultant MCP

points(occ.points.thin[[i]], col = g.colors[i], pch = 20, cex = 0.7)

plot(xy.mcp[[i]], add = TRUE, border = g.colors[i], lwd = 2)

# Saving the MCP as a .shp (Shape file)

xy.mcp[[i]] <- as(xy.mcp[[i]], "SpatialPolygonsDataFrame")

shape.directory <- paste(getwd(),'/shapes',sep="")

if (i==1){ dir.create(shape.directory) }

setwd(shape.directory)

shape.name <- paste("shape_", g.names[i], sep="")

writeOGR(xy.mcp[[i]], dsn = '.', layer = shape.name, driver = "ESRI Shapefile")

setwd(main.directory)

}

# ---------------- Preparation of the data to do the PCA ----------------

# Environmental values for the background

all.back.env <- do.call(rbind.data.frame, back.env)

# Environmental values for the species occurrence points

all.spec.env <- do.call(rbind.data.frame, spec.env)

# Environmental values all together

data.env <- rbind(all.spec.env, all.back.env)

# Saving the information of how all values are organized together in the data.env variable

# Number of rows and first row of the Species

n.rows.spec.env <- list()

first.row.spec.env <- list()

for (i in 1:n.groups){

first.row.spec.env[i] <- do.call(sum, n.rows.spec.env)+1

n.rows.spec.env[i] <- nrow(spec.env[[i]])

}

# Number of rows and first row of the Background

n.rows.back.env <- list()

first.row.back.env <- list()

for (i in 1:n.groups){

first.row.back.env[i] <- do.call(sum, n.rows.spec.env)+1+do.call(sum,n.rows.back.env)

n.rows.back.env[i] <- nrow(back.env[[i]])

}

# Rows of the data.env that correspond to the Species

rows.spec.env <- list()

for (i in 1:n.groups){

first.row <- first.row.spec.env[[i]]

last.row <- first.row.spec.env[[i]] + n.rows.spec.env[[i]]-1

rows.spec.env[[i]] <- c(first.row:last.row)

}

# Rows of the data.env that correspond to the Background

rows.back.env <- list()

for (i in 1:n.groups){

first.row <- first.row.back.env[[i]]

last.row <- first.row.back.env[[i]] + n.rows.back.env[[i]]-1

rows.back.env[[i]] <- c(first.row:last.row)

}

# ---------------- PCA ----------------

# Weight matrix

w <- c(rep(0, nrow(all.spec.env)), rep(1, nrow(all.back.env)))

# PCA of all environment

pca.cal <- dudi.pca(data.env, row.w = w, center = TRUE,

scale = TRUE, scannf = FALSE, nf = 2)

# Empty lists to save the results

scores.spec <- list()

scores.back <- list()

# Assigning the results

for(i in 1:n.groups) {

scores.spec[[i]] <- pca.cal$li[rows.spec.env[[i]], ]

scores.back[[i]] <- pca.cal$li[rows.back.env[[i]], ]

}

# All the Background results together

total.scores.back <- do.call(rbind.data.frame, scores.back)

# ---------------- Species density in the grid ----------------

#Next, we modeled the species density in the environmental grid, considering the observed occurrence density and the availability of the conditions in the background

z <- list()

for(i in 1:n.groups) {

z[[i]] <- ecospat.grid.clim.dyn(total.scores.back,

scores.back[[i]],

scores.spec[[i]],

R = R)

}

# ---------------- Niche overlap ----------------

#Niche overlap: For the niche overlap, we calculate the D metric and its significance, using a similarity test. The number of iterations has been defined at the beginning of the code

#Once the number of interactions is defined, we can generate the simulated overlap D values. Additionally, we calculate the niche dynamic indices: niche unfilling, expansion, and stability (see methods in the manuscript)

# Empty matrices

D <- matrix(nrow = n.groups, ncol = n.groups)

rownames(D) <- colnames(D) <- g.codenames

unfilling <- stability <- expansion <- sim.d <- sim.s <- eq.d <- eq.s <- D

# Filling the matrices

for(i in 2:n.groups) {

for(j in 1:(i - 1)) {

x1 <- z[[i]]

x2 <- z[[j]]

# Niche overlap

D[i, j] <- ecospat.niche.overlap (x1, x2, cor = TRUE)$D

# Niche equivalency. Note: The quantile of the environmental density used to remove marginal climates. If intersection=NA, the analysis is performed on the whole environmental extent (native and invaded). If intersection =0, the analysis is performed at the intersection between native and invaded range. If intersection=0.05, the analysis is performed at the intersection of the 5th quantile of both native and invade environmental densitie

eq.s[i, j] <- ecospat.niche.equivalency.test (x1, x2, rep, alternative = "greater")$p.I

eq.s[j, i] <- ecospat.niche.equivalency.test (x2, x1, rep, alternative = "greater")$p.I

eq.d[i, j] <- ecospat.niche.equivalency.test (x1, x2, rep, alternative = "lower")$p.I

eq.d[j, i] <- ecospat.niche.equivalency.test (x2, x1, rep, alternative = "lower")$p.I

# Niche similarity to detect more similar niches than expected by change (i.e. niche overlap is more equivalent /similar than random)

sim.s[i, j] <- ecospat.niche.similarity.test (x1, x2, rep, alternative = "greater")$p.D

sim.s[j, i] <- ecospat.niche.similarity.test (x2, x1, rep, alternative = "greater")$p.D

# Niche similarity to detect more different niches than expected by chance (i.e. the niche overlap is less equivalent/similar than random)

sim.d[i, j] <- ecospat.niche.similarity.test (x1, x2, rep, alternative = "lower")$p.D

sim.d[j, i] <- ecospat.niche.similarity.test (x2, x1, rep, alternative = "lower")$p.D

# Niche Expansion, Stability, and Unfilling

index1 <- ecospat.niche.dyn.index (x1, x2,

intersection = NA)$dynamic.index.w

index2 <- ecospat.niche.dyn.index (x2, x1,

intersection = NA)$dynamic.index.w

expansion[i, j] <- index1[1]

stability[i, j] <- index1[2]

unfilling[i, j] <- index1[3]

expansion[j, i] <- index2[1]

stability[j, i] <- index2[2]

unfilling[j, i] <- index2[3]

}

}

#Numeric results: Below, we present the complete results for each metric, considering all pair-wise comparisons between all range areas

#D value

kable(D, digits = 3, format = "markdown")

#Niche equivalency null model (p-values) to detect equivalent niches

kable(eq.s, digits = 3, format = "markdown")

#Niche equivalency null model (p-values) to detect non equivalent niches

kable(eq.d, digits = 3, format = "markdown")

#Niche similarity null model (p-values) to detect similar niches

kable(sim.s, digits = 3, format = "markdown")

#Niche similarity null model (p-values) to detect different niches

kable(sim.d, digits = 3, format = "markdown")

#Niche Unfilling:

kable(unfilling, digits = 3, format = "markdown")

#Niche Expansion:

kable(expansion, digits = 3, format = "markdown")

#Niche Stability:

kable(stability, digits = 3, format = "markdown")

# ---------------- Plots ----------------

# Function to plot the data

plot.niche.all <- function(z, n.groups, g.names,

densidade,

n.degradation,

slope.degradation,

contornar,

prob.dens,

line.width.dens,

back,

prob.back.1,

prob.back.2,

line.width.back,

title,

g.colors,

add.plots, i) {

color.vector <- function(g.colors,n.degradation,prob.dens){

alpha.seq <- numeric(length=n.degradation+1)

alpha.x <- seq(0,1,(1/n.degradation))

for (j in 1:(n.degradation+1)){

alpha.seq[j] <- (slope.degradation^alpha.x[j]-1)/(slope.degradation-1)

}

col.vector <- numeric(n.degradation+1)

current.color <- col2rgb(g.colors)/255

for (j in 1:(n.degradation+1)){

col.vector[j] <- rgb(current.color[1, ], current.color[2, ], current.color[3, ], alpha = alpha.seq[j])

}

return(col.vector)

}

a <- colorRampPalette( c(color.vector(g.colors[i], n.degradation)), alpha = TRUE )

xlim <- c(min(sapply(z, function(x){min(x$x)})),

max(sapply(z, function(x){max(x$x)})))

ylim <- c(min(sapply(z, function(x){min(x$y)})),

max(sapply(z, function(x){max(x$y)})))

z[[i]]$z.uncor@data@values <- matrix(data = z[[i]]$z.uncor@data@values, nrow = R, ncol = R, byrow = TRUE)

z[[i]]$Z@data@values <- matrix(data = z[[i]]$Z@data@values, nrow = R, ncol = R, byrow = TRUE)

image(z[[i]]$x, z[[i]]$y, z[[i]]$z.uncor@data@values, col = "transparent",

ylim = ylim, xlim = xlim,

zlim = c(0.000001, max(z[[1]]$Z@data@values, na.rm = T)),

xlab = "PC1", ylab = "PC2",cex.lab = 1.5,

cex.axis = 1.4,

add = add.plots)

abline(h = 0, v = 0, lty = 2)

box()

title(title)

if (back) {

contour(z[[i]]$x, z[[i]]$y, z[[i]]$Z@data@values, add = back,

levels = quantile(z[[i]]$Z@data@values[z[[i]]$Z@data@values > 0], probs = 1-prob.back.1),

drawlabels = FALSE,lty =1,

col = g.colors[i], lwd = line.width.back)

contour(z[[i]]$x, z[[i]]$y, z[[i]]$Z@data@values, add = back,

levels = quantile(z[[i]]$Z@data@values[z[[i]]$Z@data@values > 0], probs = 1-prob.back.2),

drawlabels = FALSE,lty =2,

col = g.colors[i], lwd = line.width.back)

}

if (densidade) {

densidade.value <- quantile(z[[i]]$z.uncor@data@values[z[[i]]$z.uncor@data@values > 0],probs = 1-prob.dens)

densidade.quantile <- z[[i]]$z.uncor@data@values

densidade.quantile[densidade.quantile < densidade.value] <- 0

densidade.quantile <- matrix(data = densidade.quantile, nrow = R, ncol = R, byrow = FALSE)

image(z[[i]]$x, z[[i]]$y, densidade.quantile, col = a(n.degradation+1), add = densidade)

}

if(contornar){

contour(z[[i]]$x, z[[i]]$y, z[[i]]$z.uncor@data@values,

add = contornar,

levels = quantile(z[[i]]$z.uncor@data@values[z[[i]]$z.uncor@data@values > 0],probs = 1-prob.dens),

drawlabels = FALSE,

col = g.colors[i],

lwd = line.width.dens, lty = 1)

}

}

# Individual niche plots

for(i in 1:n.groups) {

plot.niche.all(z, n.groups, g.names,

densidade = TRUE, # TRUE/FALSE to activate/desactivate the density plot

n.degradation = 1000, # Color resolution

slope.degradation = 100, # Color degradation constant. It has to be >1

contornar = FALSE, # TRUE/FALSE to activate/desactivate the contour of %density plot

prob.dens = 0.5, # % of the density that represents the contour (0->0% and 1->100%)

line.width.dens = 2, # Line width of the contour of %density

back = TRUE, # TRUE/FALSE to activate/desactivate the background plot

prob.back.1 = 1, # % of the background that represents the contour 1 (0->0% and 1->100%)

prob.back.2 = 0.5, # % of the background that represents the contour 2 (0->0% and 1->100%)

line.width.back = 2, # Line width of the contour of background

title = g.names[i], # Title of each region

g.colors, # Colors of the regions

add.plots = FALSE, i) # TRUE/FALSE to activate/desactivate the adition of plots in the same image

}

# Plot global Contour %Density + Occurrences Densities

for(i in 1:n.groups) {

if (i==1){ add.plots <- FALSE }

else { add.plots <- TRUE}

plot.niche.all(z, n.groups, g.names,

densidade = TRUE, # TRUE/FALSE to activate/desactivate the density plot

n.degradation = 1000, # Color resolution

slope.degradation = 100, # Color degradation constant. It has to be >1

contornar = TRUE, # TRUE/FALSE to activate/desactivate the contour of %density plot

prob.dens = 0.2, # % of the density that represents the contour (0->0% and 1->100%)

line.width.dens = 2, # Line width of the contour of %density

back = FALSE, # TRUE/FALSE to activate/desactivate the background plot

prob.back.1 = 1, # % of the background that represents the contour 1 (0->0% and 1->100%)

prob.back.2 = 0.5, # % of the background that represents the contour 2 (0->0% and 1->100%)

line.width.back = 2, # Line width of the contour of background

title = "Densities", # Title of the plot

g.colors, # Colors of the regions

add.plots = add.plots, i) # TRUE/FALSE to activate/desactivate the adition of plots in the same image

}

# Plot global Contour %Density + Occurrences Densities + Background climate

for(i in 1:n.groups) {

if (i==1){ add.plots <- FALSE }

else { add.plots <- TRUE}

plot.niche.all(z, n.groups, g.names,

densidade = TRUE, # TRUE/FALSE to activate/desactivate the density plot

n.degradation = 1000, # Color resolution

slope.degradation = 100, # Color degradation constant. It has to be >1

contornar = TRUE, # TRUE/FALSE to activate/desactivate the contour of %density plot

prob.dens = 0.98, # % of the density that represents the contour (0->0% and 1->100%)

line.width.dens = 1, # Line width of the contour of %density

back = TRUE, # TRUE/FALSE to activate/desactivate the background plot

prob.back.1 = 0.98, # % of the background that represents the contour 1 (0->0% and 1->100%)

prob.back.2 = FALSE, # % of the background that represents the contour 2 (0->0% and 1->100%)

line.width.back = 3, # Line width of the contour of background

title = "Densities and Background", # Title of the plot

g.colors, # Colors of the regions

add.plots = add.plots, i) # TRUE/FALSE to activate/desactivate the adition of plots in the same image

}

# Loadings plot

#Below the loadings plot (contribution of the variables for each axis). Check the variable codes at http://www.worldclim.org/bioclim

loadings <- cbind(cor(data.env, pca.cal$tab[,1]), cor(data.env, pca.cal$tab[,2]))

colnames(loadings) <- c("axis1", "axis2")

# Plot of the contribution of each variable to the PC1

barplot(loadings[,1], las=2, main="PC1")

# Plot of the contribution of each variable to the PC2

barplot(loadings[,2], las=2, main="PC2")

# Arrows plot

# Plot of the arrows representing the contribution of each variable, directly on the environmental space

# Get data from PCA

contrib <- pca.cal$co

eigen <- pca.cal$eig

# Preparation of the names of each variable

nomes <- gsub(paste(variables.path,'/', sep=""),'',list.variables)

nomes <- gsub(variables.file.type,'',nomes)

# Plot of the circle

s.corcircle(contrib[, 1:2] / max(abs(contrib[, 1:2])), grid = F, label = nomes, clabel = 1.2)

# Addition of the axis information

text(0, -1.1, paste("PC1 (", round(eigen[1]/sum(eigen)*100,2),"%)", sep = ""))

text(1.1, 0, paste("PC2 (", round(eigen[2]/sum(eigen)*100,2),"%)", sep = ""), srt = 90)
